# Structure-Activity Relationships of the Human Immunodeficiency Virus Type 1 Maturation Inhibitor PF-46396

**DOI:** 10.1128/JVI.01075-16

**Published:** 2016-08-26

**Authors:** Christopher Murgatroyd, Lisa Pirrie, Fanny Tran, Terry K. Smith, Nicholas J. Westwood, Catherine S. Adamson

**Affiliations:** a School of Medicine, University of St Andrews, St Andrews, Fife, United Kingdom; b School of Chemistry, University of St Andrews, St Andrews, Fife, United Kingdom; c School of Biology, University of St Andrews, St Andrews, Fife, United Kingdom; d Biomedical Sciences Research Complex, University of St Andrews, St Andrews, Fife, United Kingdom; University of Utah

## Abstract

HIV-1 maturation inhibitors are a novel class of antiretroviral compounds that consist of two structurally distinct chemical classes: betulinic acid derivatives and the pyridone-based compound PF-46396. It is currently believed that both classes act by similar modes of action to generate aberrant noninfectious particles via inhibition of CA-SP1 cleavage during Gag proteolytic processing. In this study, we utilized a series of novel analogues with decreasing similarity to PF-46396 to determine the chemical groups within PF-46396 that contribute to antiviral activity, Gag binding, and the relationship between these essential properties. A spectrum of antiviral activity (active, intermediate, and inactive) was observed across the analogue series with respect to CA-SP1 cleavage and HIV-1 (NL4-3) replication kinetics in Jurkat T cells. We demonstrate that selected inactive analogues are incorporated into wild-type (WT) immature particles and that one inactive analogue is capable of interfering with PF-46396 inhibition of CA-SP1 cleavage. Mutations that confer PF-46396 resistance can impose a defective phenotype on HIV-1 that can be rescued in a compound-dependent manner. Some inactive analogues retained the capacity to rescue PF-46396-dependent mutants (SP1-A3V, SP1-A3T, and CA-P157S), implying that they can also interact with mutant Gag. The structure-activity relationships observed in this study demonstrate that (i) the *tert*-butyl group is essential for antiviral activity but is not an absolute requirement for Gag binding, (ii) the trifluoromethyl group is optimal but not essential for antiviral activity, and (iii) the 2-aminoindan group is important for antiviral activity and Gag binding but is not essential, as its replacement is tolerated.

**IMPORTANCE** Combinations of antiretroviral drugs successfully treat HIV/AIDS patients; however, drug resistance problems make the development of new mechanistic drug classes an ongoing priority. HIV-1 maturation inhibitors are novel as they target the Gag protein, specifically by inhibiting CA-SP1 proteolytic cleavage. The lack of high-resolution structural information of the CA-SP1 target in Gag has hindered our understanding of the inhibitor-binding pocket and maturation inhibitor mode of action. Therefore, we utilized analogues of the maturation inhibitor PF-46396 as chemical tools to determine the chemical components of PF-46396 that contribute to antiviral activity and Gag binding and the relationship between these essential properties. This is the first study to report structure-activity relationships of the maturation inhibitor PF-46396. PF-46396 is chemically distinct from betulinic acid-derived maturation inhibitors; therefore, our data provide a foundation of knowledge that will aid our understanding of how structurally distinct maturation inhibitors act by similar modes of action.

## INTRODUCTION

HIV-1 infectivity is dependent upon proteolytic maturation, which occurs concomitantly with immature virus particle assembly and release from the host cell plasma membrane. Immature HIV-1 particles are assembled via multimerization of the Gag (Pr55^Gag^) protein. Gag multimerization also drives the incorporation of the Gag-Pol polyprotein that contains the viral enzymatic proteins protease (PR), reverse transcriptase (RT), and integrase (IN). Key regions of Gag that drive multimerization are the capsid (CA) C-terminal domain (CTD) and the adjoining SP1 region. More specifically, within the CA CTD, residues W184 and M185 in helix 9 are important for Gag dimerization (1, 2); while the major homology region (MHR) is required for the formation and stabilization of the Gag lattice (1–4), the CA CTD tail and the adjoining SP1 region are important for higher-order Gag-Gag multimerization (5–9). During proteolytic maturation, Gag and Gag-Pol are cleaved into their individual proteins by the virus-encoded PR. Pol is cleaved into PR, RT, and IN, and Gag is cleaved into four proteins, MA (matrix), CA, NC (nucleocapsid), and p6, and two spacer peptides, SP1 and SP2. Gag cleavage is kinetically controlled by a differential rate of processing at each of the five cleavage sites and hence follows a sequential cascade (10–15). An initial cleavage event creates an N-terminal MA-CA-SP1 fragment and a C-terminal NC-SP2-p6 fragment. These fragments are subsequently cleaved at the MA-CA and the SP2-p6 sites and finally at the NC-SP2 and CA-SP1 sites. Upon the completion of the Gag proteolytic processing cascade, the virus particle undergoes a morphological rearrangement that results in a mature infectious particle that contains the conical capsid. HIV-1 assembly, release, and proteolytic maturation have been extensively reviewed (16–20).

Correct proteolytic processing of Gag is essential for the formation of infectious HIV-1 particles, as mutations that disrupt the cleavage of individual sites or alter the order in which sites are cleaved result in aberrant particles that have significantly reduced infectivity (13, 15, 21–26). The vital role of proteolytic maturation in the production of infectious HIV-1 particles has made it an attractive target for therapeutic intervention (20, 27). Protease inhibitors (PIs) have been developed, which target HIV-1 PR enzyme activity, preventing all cleavage events in Gag and Gag-Pol and resulting in noninfectious immature virus particles. PIs have been highly successful and are routinely used in the clinic to treat HIV-1-infected patients as a component of HAART (highly active antiretroviral therapy). HAART is a combination of three to four different antiretroviral drugs and successfully reduces morbidity and mortality in HIV-1-infected patients (28–31). Combinational therapy is used due to the ease with which HIV-1 can acquire drug resistance; however, despite the tangible success of this approach, drug resistance remains an important ongoing issue that makes the identification and development of new mechanistic drug classes ongoing priorities, particularly in the face of the continued lack of an effective vaccine or cure (32).

HIV-1 maturation inhibitors are members of one such new mechanistic class of antiretroviral compounds that are under investigation (17, 32). Maturation inhibitors differ from PIs as they target the Gag substrate of PR rather than the enzymatic activity of PR. The principal mechanism of action of maturation inhibitors is the inhibition of SP1 cleavage from the C terminus of CA during the Gag proteolytic processing cascade (4, 33–35). The consequence of blocking CA-SP1 cleavage is the production of noninfectious particles with an aberrant morphology (4, 15, 33). This aberrant morphology is associated with the incomplete formation of the protein shell with a hexagonal honeycomb CA lattice, which is reminiscent of the Gag lattice in immature virus particles (36, 37). The structural organization of maturation inhibitor-treated particles suggests that, in addition to preventing CA-SP1 cleavage, maturation inhibitors also stabilize the immature Gag lattice and that both of these modes of action may contribute to the generation of noninfectious particles and, hence, the inhibitory activity of maturation inhibitors (36, 37).

Two structurally distinct chemical classes of maturation inhibitors have been identified: (i) betulinic acid derivatives, including the first-in-class compound bevirimat (BVM), also known as 3-*O*-(3′,3′-dimethylsuccinyl)betulinic acid (DSB) or PA-457 (33, 34, 38, 39), and (ii) a pyridone-based compound, PF-46396 ({1-[2-(4-tert-butylphenyl)-2-(2,3-dihydro-1H-inden-2-ylamion)ethyl]-3-(trifluoromethyl)pyridin-2(1H)-one}) (35). Despite the structural differences between these two compound classes, they both appear to act via the same general mode of action, as described above. PF-46396 has not been developed clinically, but BVM progressed to phase II clinical trials. In these trials, BVM significantly reduced the viral load in approximately half of the patients treated (40, 41). However, problems were encountered in the treatment of the remaining patients due to preexisting SP1 polymorphisms that conferred intrinsic resistance to BVM, resulting in patients failing to respond (41–45). Development of BVM was discontinued in 2010; however, potent BVM analogues that are active against HIV-1 strains that harbor SP1 polymorphisms are being developed (46–48). It is expected that these new maturation inhibitors will enable further clinical testing of this novel class of antiretroviral compounds (17).

Future development of maturation inhibitors will also benefit from further elucidation of (i) the undefined inhibitor-binding pocket in assembled Gag, (ii) the precise mode of action and mechanism(s) of inhibitor resistance, and (iii) how chemically distinct betulinic acid-derived and pyridone-based compounds apparently act via similar modes of action. Traditionally, these issues could be resolved by obtaining atomic-resolution structures of a compound bound to its target. Unfortunately, this information is not currently available for maturation inhibitors, primarily because the CA-SP1 region of Gag is disordered, as determined by X-ray crystallographic studies (1, 49), nor is it fully resolved by cryo-electron microscopy (EM)/tomography, which generates subnanometer-resolution structures of the Gag lattice in immature particles (50). However, it is broadly accepted from the available evidence that this region of Gag adopts a helical structure that exists as a hollow six-helix bundle that is situated directly below the hexagonal honeycomb CA lattice in immature particles (6, 21, 50–55). Importantly, virus assembly is required for BVM activity; hence, it is hypothesized that the unknown inhibitor-binding pocket is formed upon Gag-Gag multimerization during virus assembly (33, 56, 57). Inhibitor binding studies have shown that BVM directly interacts with two regions of Gag: the CA-SP1 junction and the upstream MHR in CA (58). To date, no direct binding studies with PF-46396 have been reported; however, competition assays with PF-46396 and BVM demonstrated that the two compounds exhibit mutually antagonistic behaviors, suggesting that these two compounds at least partially share the same binding pocket (4).

Genetic studies determining acquisition of inhibitor resistance *in vitro* have also been used to elucidate the maturation inhibitor mode of action and the residues involved in inhibitor binding. In these studies, resistance to BVM is attained by single-amino-acid mutations that map exclusively to the CA-SP1 junction of Gag (33, 34, 39, 59, 60). The preexisting SP1 polymorphisms that confer intrinsic BVM resistance (41–45) have also been mapped to this junction. In contrast, PF-46396 resistance is not conferred by the key SP1 polymorphism V7A (4). Instead, acquired resistance to PF-46396 has been mapped to mutations in the CA-SP1 junction; many of these mutations are the same as those acquired in the presence of BVM (4). Additionally, PF-46396 resistance was also acquired at CA residue 201 and the MHR that lies upstream of the CA-SP1 junction (4, 35). In the majority of cases, mutations conferring resistance to BVM were found to impair the ability of the inhibitor to bind to immature virus particles (61). This mechanism of resistance is not consistent with the BVM resistance mutation SP1-A3V, which imposes a replication defect on the virus that is rescued in a compound-dependent manner, indicating that the compound is still capable of binding to Gag in this mutant context (39). PF-46396 is also capable of rescuing SP1-A3V in a compound-dependent manner, along with other inhibitor resistance mutations that map to the CA-SP1 junction (4). Interestingly, the PF-46396 resistance mutations that map to the MHR also impose a severe replication defect on the virus, which is rescued in a compound-dependent manner by PF-46396 but not BVM (4). Rescue of these MHR mutants can also be achieved by second-site compensatory mutations that map to SP1 (4). A recent study of one of these second-site compensatory mutations, SP1-T8I, demonstrated that this mutation impairs CA-SP1 processing and stabilizes the immature Gag lattice, and hence, the phenotype of this mutant mimics the effect of maturation inhibitors (62). The evidence generated to date suggests that there is structural and functional cross talk between the CA MHR and SP1 and that BVM and PF-46396 interact differentially with a putative pocket that involves both of these regions of Gag (4).

In this study, we further investigate the mode of action/binding of PF-46396, which, since its discovery in 2009 (35), has been the subject of only two studies (4, 37). Our approach takes advantage of the synthetic tractability of this compound to generate a series of analogues that were utilized as chemical tools to further understand the mode of action of PF-46396. Our intention here was not to increase compound potency and/or drug-like properties, as Pfizer, which discovered PF-46396, has already embarked on such a campaign and reported a correlation between increased potency and lipophilicity, which is undesirable for drug development (35). Although Pfizer stated that its analogue series exhibits a definable structure-activity relationship, no details have been reported (35). Here we chemically synthesized a series of 15 analogues with decreasing similarity to the parental PF-46396 compound and report their structure-activity relationships with respect to antiviral activity, Gag binding, and the correlation between these two important properties.

## MATERIALS AND METHODS

### Cell culture, plasmids, and transfections.

HeLa cells (ATCC) were maintained in Dulbecco modified Eagle medium supplemented with 10% (vol/vol) fetal bovine serum (FBS). The Jurkat T cell line (ATCC) was maintained in RPMI 1640 supplemented with 10% (vol/vol) FBS. All media were supplemented with 2 mM l-glutamine. Plasmids used were the infectious HIV-1 molecular clone pNL4-3 (63); two derivatives with point mutations at SP1 residue 3 (SP1-A3V and SP1-A3T) (39); four derivatives with point mutations in CA at residues 156 (CA-G165E), 157 (CA-P157S), 160 (CA-P160L), and 225 (CA-G225D) (kind gift from Eric Freed, NIH, USA) (4); and two protease-defective derivatives, pNL4-3/PR^−^ with the active-site mutation PR-D25N, which contains either wild-type (WT) Gag or the SP1-A3V mutation (64). Plasmid DNA was purified with a plasmid purification maxiprep kit (Qiagen) and adjusted to 1 μg/μl. HeLa cells were transfected by using Lipofectamine 2000 (Invitrogen) according to the manufacturer's instructions, and Jurkat T cells were transfected by using DEAE-dextran (65).

### Maturation inhibitors and cellular cytotoxicity assay.

The HIV-1 maturation inhibitor PF-46396 and an inactive analogue, PF-4348182, were provided through the Pfizer Compound Transfer Program. A series of experimental PF-46396 analogues were synthesized (see Fig. S1 in the supplemental material for details of the synthetic route used and compound analysis). All compounds were dissolved in dimethyl sulfoxide (DMSO) and used at the concentrations indicated. Inhibitor cytotoxicity assays were performed by using the CellTiter 96 AQueous One Solution Cell Proliferation [3-(4,5-dimethylthiazol-2-yl)-5-(3-carboxymethoxyphenyl)-2-(4-sulfophenyl)-2H-tetrazolium (MTS)] assay (Promega) with both the HeLa and Jurkat T cell lines.

### Replication kinetics.

Jurkat T cells were transfected with pNL4-3 WT and mutant derivatives. Compounds (5 μM) were added at the time of transfection and were maintained throughout the course of the experiment. The transfected Jurkat T cells were split every Monday, Wednesday, and Friday; supernatants were collected at each time point; and viral replication was monitored by RT activity as previously described (66). Cell pellets were harvested on the days of peak RT activity, and genomic DNA was extracted by using a whole-blood DNA extraction kit (Qiagen). Maintenance of SP1 or CA mutations and acquisition of additional mutations were investigated by amplification of the entire Gag-PR coding region using forward and reverse primers NL516F (5′-TGC CCG TCT GTT GTG TGA CTC-3′) and NL2897R (5′-AAA ATA TGC ATC GCC CAC AT-3′), respectively. The resultant 2.3-kb PCR product was purified by using a QIAquick PCR purification kit (Qiagen) and then sequenced by using primers NL1410F (5′-GGA AGC TGC AGA ATG GGA TA-3′) and NL1754F (5′-TGG TCC AAA ATG CGA ACC-3′).

### Radioimmunoprecipitation analysis.

Radioimmunoprecipitation analysis was used to examine Gag proteolytic processing and virus release efficiency. Compounds were used throughout the experiment at 5 μM or the indicated concentrations and were prepared immediately prior to use and mixed by vortexing. Metabolic labeling of HeLa cells, preparation of cell and virus lysates, and immunoprecipitation methods were described in detail previously (67). In brief, HeLa cells were transfected with the pNL4-3 WT or a mutant derivative. Transfected HeLa cells were starved in Cys-Met-free medium for 30 min and then metabolically radiolabeled for 2 h with [^35^S]Cys-Met Promix (PerkinElmer). Ultracentrifugation was used to pellet virions. Cell and virus lysates were immunoprecipitated with pooled immunoglobulin from HIV-1-infected patients (HIV-Ig) obtained through the NIH AIDS Research and Reference Reagent Program, Division of AIDS, NIAID. The radioimmunoprecipitated proteins were separated by sodium dodecyl sulfate-polyacrylamide gel electrophoresis (SDS-PAGE) and exposed to X-ray film and a phosphorimager plate (Fuji), and the bands were quantified by using a Fujifilm FLA-5000 phosphorimager and ImageStudio software (Li-Cor).

### Mass spectrometric analysis to quantify compound incorporation into immature virus particles.

Immature particles were generated by transfecting HeLa cells with pNL4-3/PR^−^ or pNL4-3/PR^−^/SP1-A3V and culturing the cells in the presence of the compound at 5 μM. Virus-containing supernatants were harvested, clarified by centrifugation for 5 min at 1,200 rpm at 4°C, and then filtered by using a 0.45-μm filter disc. Particles were concentrated by ultracentrifugation through a 20% sucrose cushion at 100,000 × *g* for 2 h at 4°C. Pelleted virus particles were suspended in STE buffer (100 mM NaCl, 50 mM Tris-HCl, 1 mM EDTA [pH 7.4]) and purified by equilibrium ultracentrifugation on a linear 30 to 70% sucrose gradient for 16 h at 36,000 rpm at 4°C in a Beckman SW55 rotor. Fractions of 0.5 ml were harvested and assayed for the presence of Gag via SDS-PAGE and Western blotting, followed by immunodetection with HIV-Ig and goat anti-human IRDye680RD-conjugated secondary antibody (Li-Cor). Peak fractions were pooled, and total Gag was quantified by using the same SDS-PAGE/Western blot/immunodetection procedure and scanned by using an Odyssey CLx instrument (Li-Cor). To quantify the levels of the compound present, pooled fractions were spiked with 100 pmol of StA-MAT-5 as an internal standard prior to being extracted with a 3:1 (vol/vol) solution of chloroform and methanol. The chloroform-rich phase containing the extracted analogues and the internal standard was transferred to a new glass tube and dried under a flow of nitrogen gas. Samples were suspended in 15 μl of a 1:2 (vol/vol) solution of chloroform-methanol and 5 μl of acetonitrile-isopropanol-water (6:7:2) and delivered using a NanoMate instrument (Advion) to an AB Sciex 4000 quadrupole ion-trap mass spectrometry instrument with a nanoelectrospray source, using nitrogen as the collision gas. A multiple-reaction monitoring (MRM) approach was utilized to quantify the compound bound to the isolated virus particles. The MRM mass transition (Table 1) for each of the compounds was determined in the positive-ion mode (entrance potential [EP] of 8 eV, collision cell exit potential [CXP] of 12 eV, interface temperature of 30°C, gas pressure of 0.5 lb/in^2^, tip voltage of 1.25 to 1.5 kV, and dwell time of 500 ms), and spectra were acquired for 2 min. All MRM data were normalized to the internal standard before standard curves were generated (0.1 to 500 pmol) for each of the compounds, allowing its own response factor to be determined. Samples were analyzed in the same manner, allowing quantification of the compounds extracted from the isolated virus particles.

**TABLE 1:** MRM transitions^*a*^

Metabolite	MRM transition (m/z)	Collision energy (V)	Declustering potential (V)	Response factor
StA-MAT-5 (IS)	338→147	50	45	50.63
PF-46396	455→322	40	40	58.69
PF-4348182	438→305	25	35	45.85
StA-MAT-7	338→180	50	40	72.66
StA-MAT-23	413→260	40	40	94.88
StA-MAT-30	435→322	45	40	112.90

a IS, internal standard.

## RESULTS

### Synthesis of a novel analogue series based on the HIV-1 maturation inhibitor PF-46396.

**FIG 1:**
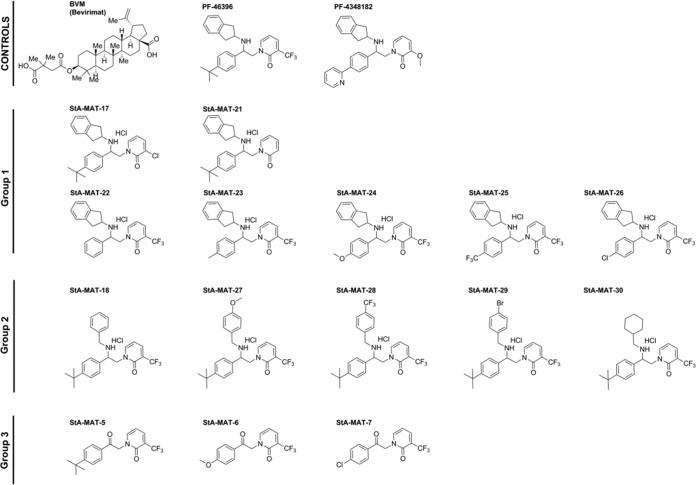
Chemical structures of BVM, PF-46396, and analogues. Controls are the maturation inhibitors BVM, PF-46396, and PF-4348182, which is an inactive analogue with a replacement of the *tert*-butyl group on the phenyl ring with a 2-pyridyl group and a replacement of the trifluoromethyl group on the pyridone core with a methoxy group. The test analogue series consisted of three groups. Group 1 analogues had a replacement of the trifluoromethyl group on the pyridone core with a chloro group (StA-MAT-17) or no substituent (StA-MAT-21) or a replacement of the *tert*-butyl group on the phenyl ring with no substituent (StA-MAT-22), a methyl group (StA-MAT-23), a methoxy group (StA-MAT-24), a trifluoromethyl group (StA-MAT-25), or a chloro group (StA-MAT-26). Group 2 analogues had the 2-aminoindan group replaced with a benzylamine fragment with no substituent on the benzyl ring (StA-MAT-18) or with a replacement at position 4 of the benzyl ring with a methoxy group (StA-MAT-27), a trifluoromethoxy group (StA-MAT-28), or a bromo group (StA-MAT-29) or had the 2-aminoindan group replaced with an (aminomethyl)cyclohexane group (StA-MAT-30). Group 3 contains analogues with no 2-aminoindan group (StA-MAT-5) or no 2-aminoindan group and a replacement of the *tert*-butyl group on the phenyl ring with a methoxy group (StA-MAT-6) or a chloro group (StA-MAT-7).

In order to further interrogate the mode of action of known HIV-1 maturation inhibitors, we synthesized an initial analogue series based on the second-in-class pyridone-based compound PF-46396 (Fig. 1). The analogue series can be broadly divided into 3 groups, group 1, group 2, and group 3, which have decreasing similarity to the parental compound PF-46396. In group 1, the 2-aminoindan group is unchanged, but modifications are introduced to the trifluoromethyl group on the pyridone core (StA-MAT-17 and -21) or the *tert*-butyl group on the phenyl ring (StA-MAT-22, -23, -24, -25, and -26). In group 2, the 2-aminoindan group was replaced with a benzylamine fragment with various substituents (StA-MAT-18, -27, -28, -29, and -30), and group 3 represents precursor ketones without the 2-aminoindan group (StA-MAT-5, -6, and -7).

The novel PF-46396 analogues were synthesized by using an efficient 3-step sequence from commercially available starting materials (see Fig. S1 and associated experimental procedures in the supplemental material). All analogues were shown to be of the required structure and purity level by using a range of analytical methods (see Fig. S1 in the supplemental material for details). Compound cytotoxicity was measured by using an MTS assay to monitor cell viability via cellular redox potential. PF-46396, PF-4348182, and the analogue series at concentrations of ≤10 μM did not cause cytotoxicity after 24 h in the HeLa cell line or Jurkat T cells (data not shown). All subsequent experiments were performed with compounds at a final concentration of 5 μM, and no significant toxicity was observed. The only exception was incubation with 5 μM StA-MAT-18, -24, and -30 for >7 days in Jurkat T cells, where some cellular cytotoxicity was observed by microscopy; however, HIV-1 replication was not inhibited.

### Effect of the PF-46396 analogue series on inhibition of CA-SP1 processing and HIV-1 replication.

**FIG 2:**
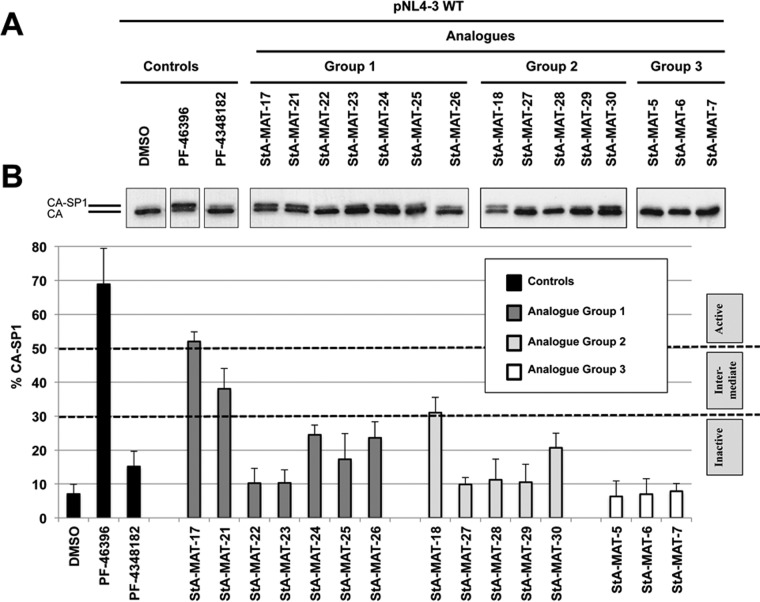
Inhibition of CA-SP1 processing by the PF-46396 analogue series as determined by a quantitative CA-SP1 processing assay. HeLa cells were transfected with pNL4-3/WT and cultured with 5 μM compound or an equivalent volume of DMSO. Cells were metabolically labeled for 2 h with [^35^S]Met-Cys, and released virions were pelleted by ultracentrifugation. (A) Virus lysates were immunoprecipitated with HIV-Ig, and processing of CA-SP1 to CA was analyzed by SDS-PAGE and fluorography. (B) Phosphorimager analysis was used to quantify the percentage of CA-SP1 relative to total CA-SP1 plus CA in pelleted virus samples. Mean values from independent experiments are presented (*n* = 3), and error bars indicate standard deviations.

The principal antiviral mode of action of HIV-1 maturation inhibitors is the inhibition of CA-SP1 proteolytic processing, the consequence of which is the accumulation of an uncleaved CA-SP1 intermediate and the formation of aberrant noninfectious virus particles (4, 33–35, 38). We tested analogue activity by examining the inhibition of CA-SP1 proteolytic cleavage using a previously described biochemical assay to quantitate CA-SP1 levels in virus particles (39). The assay was performed by using the wild-type pNL4-3 HIV-1 infectious molecular clone and either the required volume of a compound stock solution in DMSO to give a final compound concentration of 5 μM or an equivalent volume of DMSO (Fig. 2). Consistent with previously reported results (4, 35), treatment with PF-46396 resulted in a significant accumulation of the uncleaved CA-SP1 intermediate in virus particles. A spectrum of CA-SP1 processing-inhibitory activity was observed across the analogue series, and analogues were categorized as being either active (≥50% CA-SP1), intermediate (30 to 50% CA-SP1), or inactive (≤30% CA-SP1). These designations are derived from previous studies correlating variable levels of uncleaved CA-SP1 and effects on HIV-1 infectivity (26, 43). Only one analogue, StA-MAT-17 from group 1, was designated active; however, its activity was reduced compared to that of PF-46396. Two analogues, StA-MAT-21 and StA-MAT-18 from group 1 and group 2, respectively, exhibited intermediate inhibition of CA-SP1 proteolytic processing. All the other analogues were designated inactive. A subset of these inactive analogues (PF-4348182 and StA-MAT-24, -25, -26, and -30) generated a small amount of the uncleaved CA-SP1 intermediate, suggesting that these analogues retain a very low level of inhibitory activity but that this is unlikely to impact virus infectivity.

**FIG 3:**
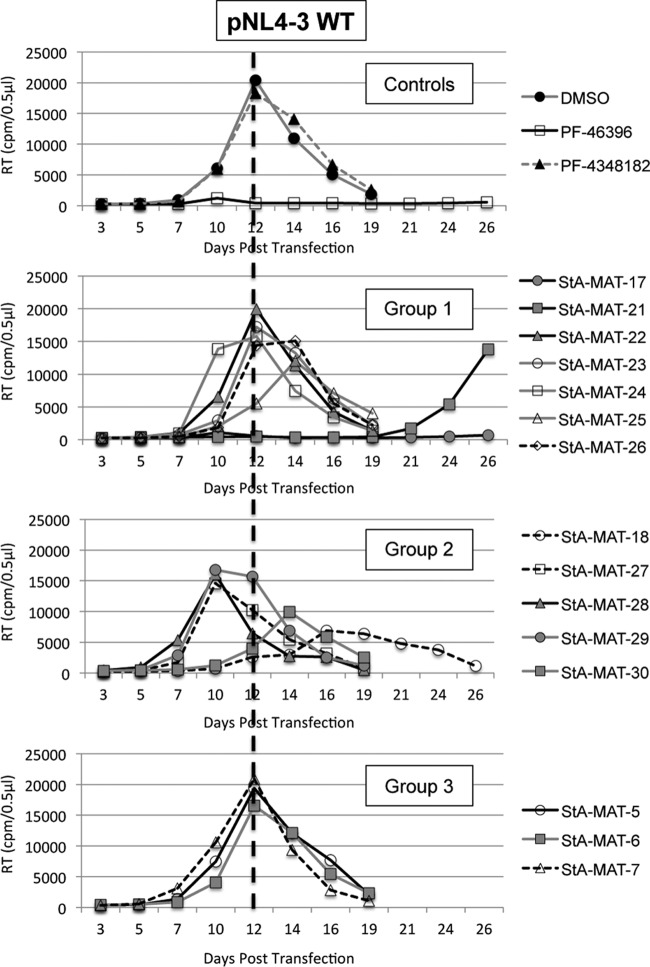
Effects of PF-46393 analogues on WT pNL4-3 HIV-1 replication kinetics in Jurkat T cells. Jurkat T cells were transfected with WT pNL4-3 and cultured with 5 μM compound or an equivalent volume of DMSO. Cells were split at regular intervals, and supernatants were reserved at each time point for RT analysis. The dashed line represents the peak day (day 12) of WT NL4-3 replication when cultured in DMSO.

To correlate the biochemical data with the effect of the analogues on HIV-1 infectivity, we evaluated virus replication kinetics in the Jurkat T cell line. The WT pNL4-3 HIV-1 infectious molecular clone was transfected into Jurkat T cells and passaged over time either in the absence of the compound or in the presence of a final compound concentration of 5 μM (Fig. 3). In the absence of the compound, WT NL4-3 replication peaked at 12 days posttransfection. As expected, the active maturation inhibitor PF-46396 completely inhibited virus replication (up to 26 days in culture, when the experiment was terminated). HIV-1 replication was not inhibited by any of the analogues except StA-MAT-17, -21, and -18. Like PA-46396, StA-MAT-17 completely suppressed virus replication for the duration of the experiment. The analogue StA-MAT-21 inhibited virus replication until 24 days posttransfection, when virus replication began to emerge. Genomic DNA was extracted from virus-infected cells, the Gag and PR coding regions were PCR amplified, and the CA CTD plus SP1 were sequenced to demonstrate that the virus had acquired the previously identified PF-46396 resistance mutation SP1-A3V (4). The delay in virus replication and acquisition of a resistance mutation demonstrates that StA-MAT-21 imposes an inhibitory activity on HIV-1 replication despite this analogue imposing intermediate (38%) inhibition of CA-SP1 cleavage. The other analogue, StA-MAT-18, that exhibited intermediate (31%) inhibition of CA-SP1 cleavage also resulted in delayed replication kinetics. Replication was slow to begin and plateaued at between 16 and 21 days posttransfection. Sequencing revealed that this virus did not acquire any mutations in the CA CTD or SP1. Therefore, StA-MAT-18 causes delayed HIV-1 replication, but it does not impose sufficient inhibitory pressure on the virus such that the acquisition of a resistance mutation is a requirement for virus replication. A repeat of this replication experiment, which used a subset of analogues, verified the observations described above.

Overall, the results obtained in the biochemical and replication data sets are in agreement. All analogues except StA-MAT-17, -21, and -18 are inactive. PF-4348182, the negative control provided by Pfizer, is included within the inactive set of analogues. Only two analogues, StA-MAT-17 and -21, have antiviral activity, which imposes sufficient pressure on the virus to result in the acquisition of a resistance mutation. StA-MAT-18 has intermediate antiviral activity but does not impose sufficient pressure on the virus to result in the acquisition of resistance mutations. The spectrum of activities across the analogue series allows us to gain insights into structure-activity relationships for this class of HIV-1 maturation inhibitors with respect to the inhibition of CA-SP1 proteolytic processing.

### Rescue of SP1 residue 3 mutants by PF-46396 analogues.

Resistance to maturation inhibitors can impose a defective phenotype on HIV-1 that can be rescued in a compound-dependent manner (4, 39). This compound dependence implies that the mechanism of resistance is not via the prevention of compound binding, but instead, the mutation causes a conformational change in Gag that permits PR access to the CA-SP1 cleavage site even when the compound is bound. In order to gain further insights into the mode of action of PF-46396, we determined if our panel of analogues retained the ability to rescue mutant HIV-1 and, hence, interact with Gag, at least in the context of the mutant tested. In the first instance, we tested the SP1 residue 3 mutants SP1-A3V and SP1-A3T. BVM was previously shown to rescue the replication of the SP1-A3V mutant but not the more severe SP1-A3T mutant (39), whereas PF-46396 rescues the replication of both SP1-A3V and SP1-A3T (4).

**FIG 4:**
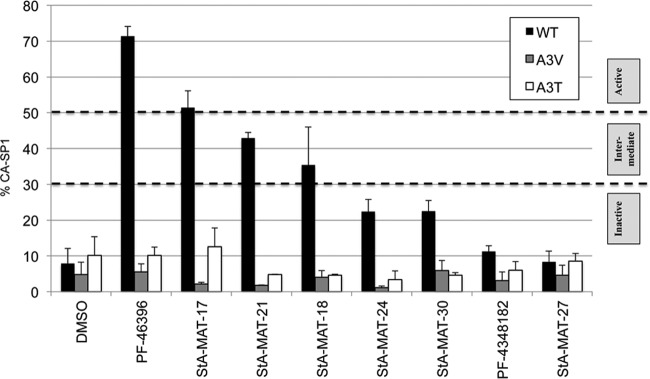
SP1 residue 3 mutations confer resistance to PF-46396 analogues. The quantitative CA-SP1 processing assay involves HeLa cells being transfected with WT pNL4-3 or derivatives containing the SP1-A3V or SP1-A3T mutation and cultured with 5 μM compound or an equivalent volume of DMSO. Cells were metabolically labeled for 2 h with [^35^S]Met-Cys, and released virions were pelleted by ultracentrifugation. Virus lysates were immunoprecipitated with HIV-Ig, and processing of CA-SP1 to CA was analyzed by SDS-PAGE and fluorography, followed by phosphorimager analysis to quantify the percentage of CA-SP1 relative to total CA-SP1 plus CA in pelleted virus samples. Mean values from independent experiments are presented (*n* = 3), and error bars indicate standard deviations.

**FIG 5:**
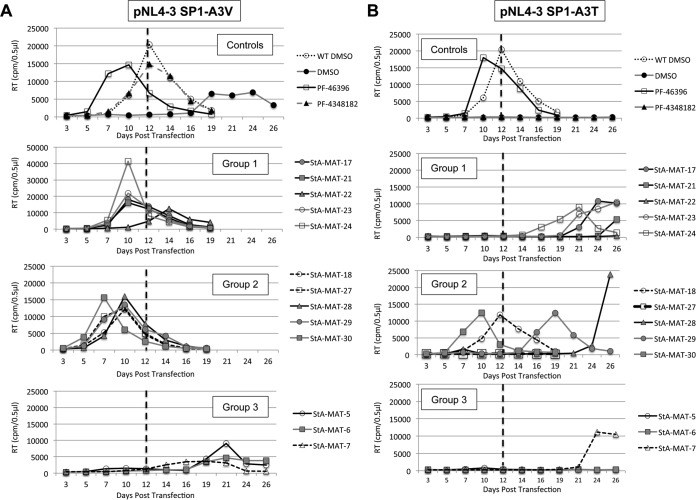
Effect of PF-46393 analogues on replication kinetics of HIV-1 SP1 residue 3 mutants in Jurkat T cells. Jurkat T cells were transfected with pNL4-3/WT, pNL4-3/SP1-A3V (A), or pNL4-3/SP1-A3T (B) and cultured with 5 μM compound or an equivalent volume of DMSO. Cells were split at regular intervals, and supernatants were reserved at each time point for RT analysis. The dashed line represents the peak day (day 12) of WT NL4-3 replication when cultured in DMSO.

We first confirmed that the SP1 residue 3 mutations conferred resistance to the analogue series by testing a selection of analogues with various degrees of inhibitory activity (Fig. 4). The ability of the analogue to rescue defective SP1-A3V and SP1-A3T replication was evaluated in the Jurkat T cell line (Fig. 5). Due to low yields of StA-MAT-25 and -26 during synthesis, these two analogues were not included in the experiment presented in Fig. 5. However, StA-MAT-25 was included in a confirmatory repeat experiment that utilized a subset of analogues (Table 2). The replication capacity of the SP1 residue 3 mutants was consistent with previously reported results (4, 39). In the absence of the compound, replication of the SP1-A3V mutant was delayed by 12 days compared to the WT virus, and the emergence of replication coincided with the acquisition of a second-site mutation (SP1-T8I) in combination with the SP1-A3V mutation (Fig. 5) or a loss of the SP1-A3V mutation and reversion to the WT sequence (Table 2). In contrast, the SP1-A3T mutant displayed no detectable replication over the course of the experiment (Fig. 5 and Table 2).

**TABLE 2:** Effects of a subset of PF-46396 analogues on HIV-1 SP1 residue 3 mutant replication kinetics in Jurkat T cells^*a*^

NL4-3 variant	Analogue	Day of peak replication compared to that with DMSO	Mutation acquisition
WT	DMSO	0	ND
SP1-A3V	DMSO	+11	Reverted to WT
SP1-A3V	PF-46396	−3	ND
SP1-A3V	PF-4348182	+2	ND
SP1-A3V	StA-MAT-21	0	ND
SP1-A3V	StA-MAT-23	0	ND
SP1-A3V	StA-MAT-25	0	ND
SP1-A3V	StA-MAT-18	−3	SP1-A3V maintained
SP1-A3V	StA-MAT-27	0	ND
SP1-A3V	StA-MAT-30	−3	SP1-A3V maintained
SP1-A3T	DMSO	No replication	ND
SP1-A3T	PF-46396	0	ND
SP1-A3T	PF-4348182	No replication	ND
SP1-A3T	StA-MAT-21	+7	SP1-A3V
SP1-A3T	StA-MAT-23	+7	SP1-A3V
SP1-A3T	StA-MAT-25	+2	SP1-A3T maintained
SP1-A3T	StA-MAT-18	0	SP1-A3T maintained
SP1-A3T	StA-MAT-27	+7	SP1-A3V
SP1-A3T	StA-MAT-30	−3	SP1-A3T maintained

a ND, not determined.

As expected, PF-46396 completely reversed the replication defect imposed by both the SP1-A3V and SP1-A3T mutations. Interestingly, PF-4348182 and all analogues belonging to groups 1 and 2 completely rescued SP1-A3V replication irrespective of their inhibitory activity against HIV-1. For example, both the active analogue StA-MAT-17 and the completely inactive analogue StA-MAT-27 rescued SP1-A3V replication to the same extent, such that virus replication peaked 2 days earlier than for the WT virus (Fig. 5). Rescue of the more severe SP1-A3T mutant was achieved only by PF-46396 and a subset of analogues belonging to groups 1 and 2 (StA-MAT-25, -18, and -30), but again, the capacity to rescue was independent of the compound's inhibitory activity against HIV-1 (Fig. 5 and Table 2). The lack of any replication delay implies that the mutants are not under any selection pressure. This was confirmed by sequencing of representative samples, which demonstrated that upon rescue, the original SP1 residue 3 mutations were maintained without the acquisition of additional mutations (Table 2). Intriguingly, in contrast to the complete lack of SP1-A3T replication in the absence of the compound, delayed replication was observed in the presence of group 1 and 2 analogues that did not rescue this mutant, and the emergence of replication was accompanied by the loss of the SP1-A3T mutation and acquisition of the SP1-A3V mutation. Conversion to SP1-A3V must offer an advantage in the presence of these analogues, as it requires 2 nucleotide changes compared to just 1 nucleotide change to revert to the WT sequence. In contrast to the across-the-board rescue of SP1-A3V observed for group 1 and 2 analogues, no group 3 analogues rescued SP1-A3V or SP1-A3T (Fig. 5). When virus replication occurred in the presence of a group 3 analogue, it was significantly delayed (+9 days), and the SP1-A3V mutation had reverted to the WT sequence.

**FIG 6:**
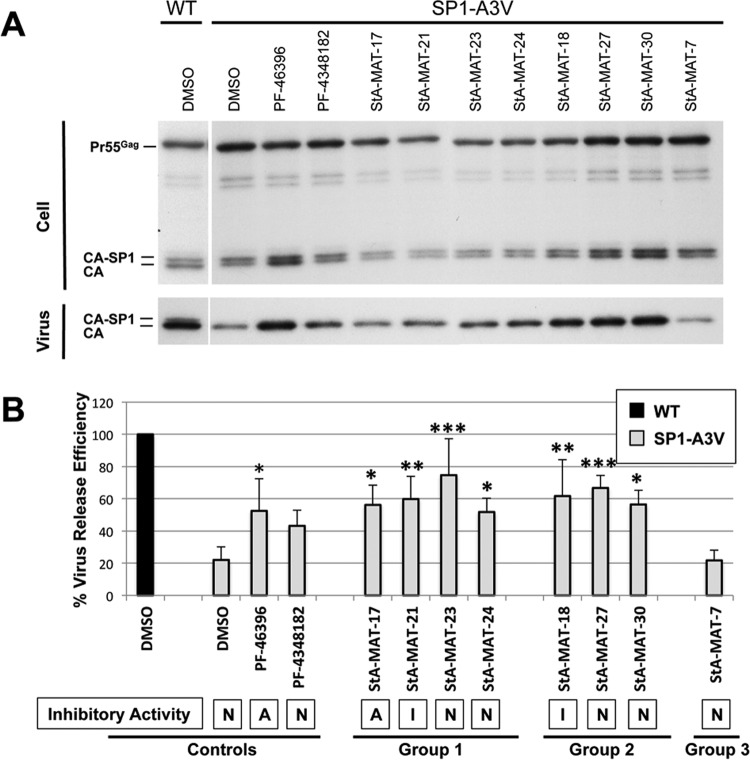
Effect of PF-46396 analogues on virus release efficiency of the SP1-A3V mutant. HeLa cells were transfected with pNL4-3/WT or pNL4-3/SP1-A3V and cultured with 5 μM compound or an equivalent volume of DMSO. Cells were metabolically labeled for 2 h with [^35^S]Met-Cys, and released virions were pelleted by ultracentrifugation. (A) Cell and virus lysates were immunoprecipitated with HIV-Ig and analyzed by SDS-PAGE and fluorography. (B) Phosphorimager analysis was done to quantify virus release efficiency, calculated as the amount of particle-associated Gag as a fraction of total (cell and virion) Gag. Statistical significance was assessed by using one-way analysis of variance followed by Dunnett's multiple-comparison posttest to compare SP1-A3V virus release efficiency in the presence of DMSO to that in the presence of each test compound. *, *P* < 0.05; **, *P* < 0.01; ***, *P* < 0.001. Mean values from independent experiments are presented (*n* = 3 to 6), and error bars indicate standard deviations. Statistical analysis was performed by using GraphPad Prism version 6 software. The compound's ability to inhibit HIV-1 is indicated (A, active; I, intermediate activity; N, nonactive).

**FIG 7:**
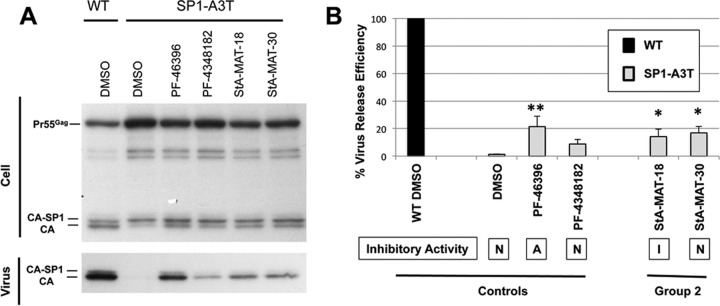
Effects of PF-46396 analogues on virus release efficiency of the SP1-A3T mutant. HeLa cells were transfected with pNL4-3/WT or pNL4-3/SP1-A3T and cultured with 5 μM compound or an equivalent volume of DMSO. Cells were metabolically labeled for 2 h with [^35^S]Met-Cys, and released virions were pelleted by ultracentrifugation. (A) Cell and virus lysates were immunoprecipitated with HIV-Ig and analyzed by SDS-PAGE and fluorography. (B) Phosphorimager analysis was used to quantify virus release efficiency, calculated as the amount of particle-associated Gag as a fraction of total (cell plus virion) Gag. Statistical significance was assessed by using one-way analysis of variance followed by Dunnett's multiple-comparison posttest to compare SP1-A3T virus release efficiency in the presence of DMSO to that in the presence of each test compound. *, *P* < 0.05; **, *P* < 0.01; ***, *P* < 0.001. Mean values from independent experiments are presented (*n* = 2 to 4), and error bars indicate standard deviations. Statistical analysis was performed by using GraphPad Prism version 6 software. The compound's ability to inhibit HIV-1 is indicated (A, active; I, intermediate activity; N, nonactive).

The SP1 residue 3 mutations cause defective virus particle assembly and release, which is again more severe in the SP1-A3T mutant (39). PF-46396 partially corrects defective virus particle production in these mutants (4); therefore, we sought to determine if a representative subset of analogues that rescued SP1-A3V or SP1-A3T replication also restored virus particle production by measuring virus release efficiency (Fig. 6 and 7). Overall, the ability of an analogue to rescue defective virus replication correlated with a partial restoration of virus particle release efficiency and was independent of the analogue's inhibitory activity.

SP1-A3V virus release efficiency was significantly increased in the presence of all the compounds tested that have the ability to rescue SP1-A3V replication, with the exception of PF-4348182. Despite the fact that treatment with PF-434812 did not result in a statistically significant increase in virus release efficiency, the data suggest that this compound likely complies with the overall trend, as the mean virus release efficiency in its presence increased to 44%, compared to 22% in the presence of DMSO (negative control). Reassuringly, the group 3 analogue StA-MAT-7, which did not rescue SP1-A3V replication, also did not restore any virus particle release efficiency. SP1-A3T virus release efficiency was significantly increased in the presence of PF-46396, StA-MAT-18, and StA-MAT-30, which rescued SP1-A3T replication. Treatment with PF-4348182, which did not rescue SP1-ATV replication, did not restore virus particle release efficiency, as no statistically significant increase was observed.

Overall, an analogue's ability to rescue virus replication correlated with a partial restoration of virus particle production that was generally equivalent to the level of restoration achieved by PF-46396. The ability of an analogue to rescue the defective phenotype conferred by the SP1 residue 3 mutations was independent of inhibitory activity against HIV-1, and rescue implies that the analogue was able to interact with the mutant Gag protein concerned.

### Rescue of CA mutants by inactive PF-46396 analogues.

**FIG 8:**
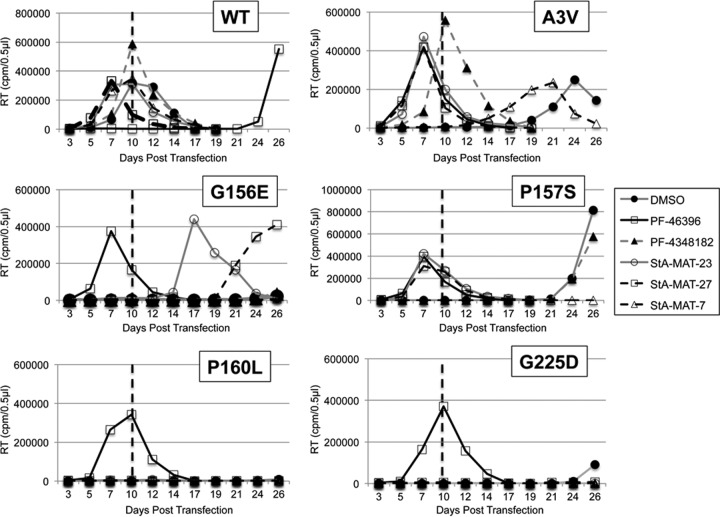
Effect of PF-46393 analogues on pNL4-3 CA mutant replication in Jurkat T cells. Jurkat T cells were transfected with pNL4-3/WT or SP1-A3V, CA-G156E, CA-157S, CA-P160L, or CA-G225D derivatives and cultured with either 5 μM compound or an equivalent volume of DMSO. Cells were split at regular intervals, and supernatants were reserved at each time point for RT analysis. The dashed line represents the peak day (day 10) of WT NL4-3 replication when cultured in DMSO.

PF-46396 resistance mutations located in CA also impose severe replication and virus production defects on HIV-1 that can be rescued in a compound-dependent manner by PF-46396 but not BVM (4). These mutations include a mutation located at the CA CTD tail (CA-G225D) and 3 mutations that map to the MHR (CA-G156E, CA-P157S, and CA-P160L). Given our observation that inactive analogues can rescue the SP1 residue 3 mutations, we investigated if a selection of our inactive analogues (PF-4348182 and StA-MAT-23, -27, and -7) could rescue these CA mutants (Fig. 8). StA-MAT-7 did not rescue the replication of any of the CA mutants; however, this was not surprising given that none of the group 3 analogues rescued the SP1 residue 3 mutants. Of the other three analogues tested that rescued SP1-A3V, only StA-MAT-23 and -27 were able to completely rescue the replication of just one CA mutant, CA-P157S. The CA-G156E mutant exhibited moderately delayed replication (+7 days) in the presence of StA-MAT-23; unfortunately, this sample was not collected for sequencing, and therefore, it is not possible to distinguish between the possibility that StA-MAT-23 is capable of partially rescuing CA-G156E replication and the possibility that the virus acquired a mutation that either reverted the virus back to the WT sequence or provided a second-site mutation that compensated for the replication defect imposed by CA-G156E. It was previously demonstrated that these CA mutants can acquire such second-site compensatory mutations (4); therefore, this is likely to have also occurred in this experiment when the replication of both CA-G156E and CA-P157S emerged after a significant delay.

**FIG 9:**
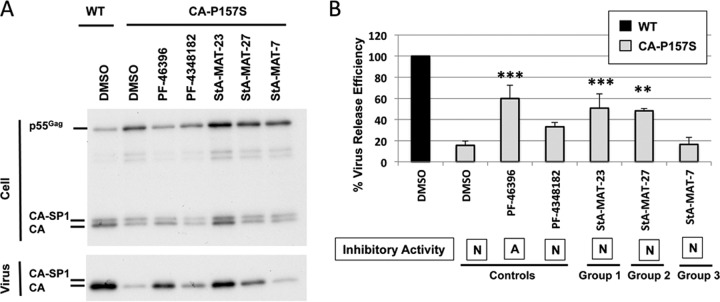
Effect of inactive PF-46396 analogues on virus release efficiency of the CA-P175S mutant. HeLa cells were transfected with pNL4-3/WT or pNL4-3/CA-P175S and cultured with 5 μM compound or an equivalent volume of DMSO. Cells were metabolically labeled for 2 h with [^35^S]Met-Cys, and released virions were pelleted by ultracentrifugation. (A) Cell and virus lysates were immunoprecipitated with HIV-Ig and analyzed by SDS-PAGE and fluorography. (B) Phosphorimager analysis was performed to quantify virus release efficiency, calculated as the amount of particle-associated Gag as a fraction of total (cell and virion) Gag. Statistical significance was assessed by using one-way analysis of variance followed by Dunnett's multiple-comparison posttest to compare CA-P157S virus release efficiency in the presence of DMSO to that in the presence of each test compound. *, *P* < 0.05; **, *P* < 0.01; ***, *P* < 0.001. Mean values from independent experiments are presented (*n* = 3), and error bars indicate standard deviations. Statistical analysis was performed by using GraphPad Prism version 6 software. The compound's ability to inhibit HIV-1 is indicated (A, active; I, intermediate activity; N, nonactive).

An analogue's ability to rescue CA-P157S replication also correlated with partial restoration of the particle production defect imposed on HIV-1 by this mutation (Fig. 9). The compounds (PF-46396 and StA-MAT-23 and -27) that rescued CA-P175S replication exhibited a statistically significant increase in CA-P175S virus release efficiency. In contrast, PF-4348182, which did not rescue CA-P175S replication, also did not cause a statistically significant increase in CA-P175S virus release efficiency. Although not all the inactive analogues tested were capable of rescuing the CA-P157S mutant, this capability was independent of antiviral activity because PF-46396 can also rescue CA-P157S along with the other CA mutants.

### Inactive PF-46396 analogues are capable of associating with WT and mutant Gag proteins in the context of immature virus particles.

We have demonstrated that some of the inactive PF-46396 analogues are capable of rescuing defective HIV-1 replication and virus particle production in a compound-dependent manner. The defective replication phenotype is imposed on HIV-1 by a variety of mutations that confer PF-46396 resistance and map to the CA-SP1 junction or MHR of Gag. The observed compound dependence strongly suggests that the relevant inactive analogues bind to mutant Gag molecules in order to facilitate rescue. We sought to further demonstrate that inactive analogues are capable of binding mutant Gag proteins by determining if inactive analogues are incorporated into immature virus particles using a variation of a previously reported mass spectrometry-based method that demonstrated BVM incorporation into immature HIV-1 particles (57). The experiment was conducted in the context of assembled virus particles, rather than using purified Gag protein, because it was previously shown that maturation inhibitor binding requires virus assembly (33, 56, 57).

**FIG 10:**
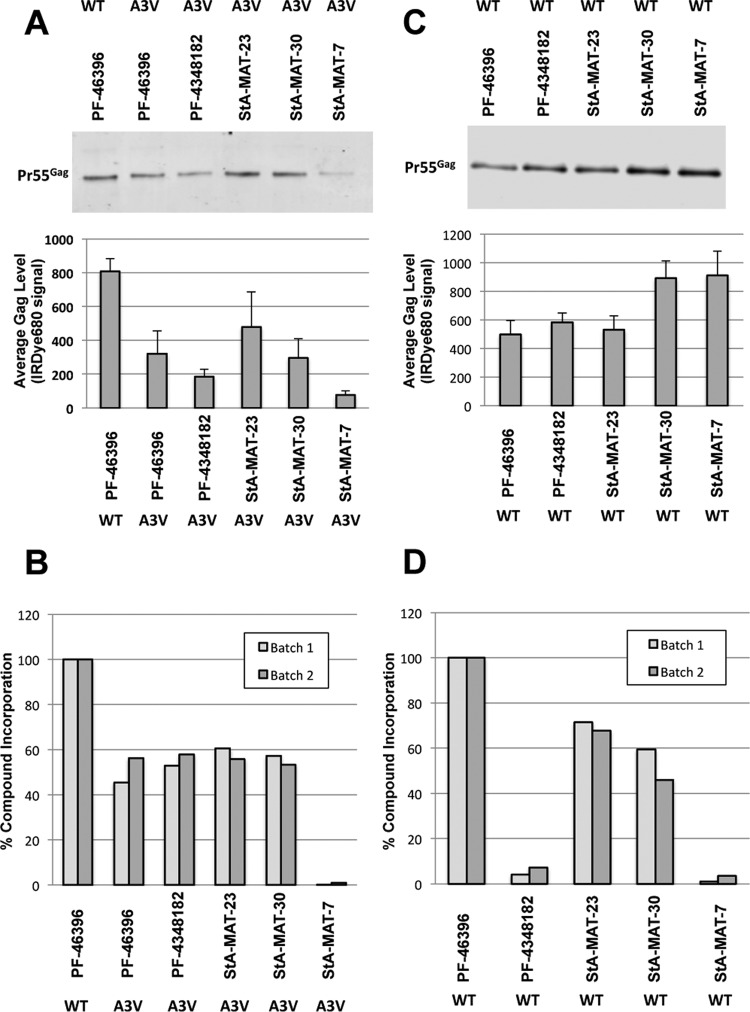
Incorporation of PF-46396 analogues into immature WT or SP1-A3V HIV-1 particles. HeLa cells were transfected with pNL4-3/PR^−^/WT or pNL4-3/PR^−^/SP1-A3V and treated with 5 μM compound. Virus particles were concentrated by ultracentrifugation through a 20% sucrose cushion and purified by equilibrium ultracentrifugation on a linear 30 to 70% sucrose gradient. (A and C) Peak fractions were pooled, and total Gag quantitated by immunodetection. Compounds in pooled peak fractions were extracted, and the amount of the compound present was quantitated by mass spectrometric analysis. (B and D) The relative incorporation of the compound was normalized to the amount of Gag in each sample and is represented as a percentage of the PF-46396 control. Batches 1 and 2 represent two independent experiments.

To conduct this experiment, we selected the SP1-A3V mutant because (i) it was rescued in a compound-dependent manner by the greatest variety of analogues and (ii) it imposes the least severe defect in virus particle production compared to the other mutants investigated (SP1-A3T and CA-P157S) and, hence, is most likely to generate sufficient particles to enable us to determine analogue incorporation. We tested the following inactive compounds: PF-4348182 and StA-MAT-23, -30, and -7 from groups 1, 2, and 3, respectively. The incorporation of PF-46396 into both WT and SP1-A3V immature particles was also determined. Immature particles were produced in the presence of the compound. Relative levels of analogue incorporation were normalized to the amount of Gag in each purified virus particle sample, and levels of PF-46396 incorporation into WT particles were set at 100% (Fig. 10).

The inactive analogues PF-4348182 and StA-MAT-23 and -30, which are capable of rescuing the replication defect imposed on HIV-1 by the SP1-A3V mutation, were incorporated into SP1-A3V immature particles to the same extent as the active compound PF-46396 (Fig. 10B). As expected, the inactive analogue StA-MAT-7, which is not capable of rescuing SP1-A3V virus replication, was not incorporated into SP1-A3V immature particles (Fig. 10B). Interestingly, all the compounds that were incorporated into SP1-A3V immature particles were present at a level that was approximately half of that observed for PF-46396 incorporation into WT immature virus particles (Fig. 10B). Overall, these data support the hypothesis that inactive compounds are capable of interacting with mutant Gag proteins in order to rescue defective HIV-1 in a compound-dependent manner.

The observed ability of inactive compounds to interact with mutant Gag may be a consequence of a conformational change in Gag, caused by the mutations, which could modify either the compound binding site and/or binding affinity. Therefore, it cannot necessarily be assumed that inactive compounds can interact with WT Gag. We used a mass spectrometry assay to determine if the same inactive analogues are also incorporated into WT immature particles (Fig. 10C and D). As anticipated, the group 3 analogue StA-MAT-7 was not incorporated into WT immature particles, suggesting that this analogue has lost its ability to interact with Gag. PF-4348182 was detected at very low levels in WT immature virus particles, suggesting that this compound interacts only weakly, if at all, with WT Gag. This demonstrates that a compound's ability to bind mutant Gag cannot be directly extrapolated to infer an ability to bind WT Gag. The inactive analogues StA-MAT-23 and -30, however, were incorporated into WT particles albeit at reduced levels compared to PF-46396. The incorporation of StA-MAT-23, however, is remarkably efficient given that this analogue exhibits absolutely no inhibitory activity against HIV-1.

**FIG 11:**
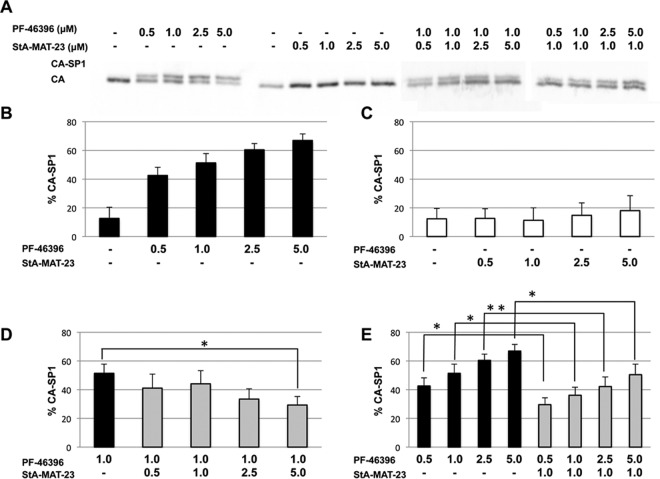
The inactive analogue StA-MAT-23 interferes with PF-46396 activity. HeLa cells were transfected with pNL4-3/WT and cultured with the indicated concentrations of PF-46396 and/or StA-MAT-23. Cells were metabolically labeled for 2 h with [^35^S]Met-Cys, and released virions were pelleted by ultracentrifugation. (A) Virus lysates were immunoprecipitated with HIV-Ig, and processing of CA-SP1 to CA was analyzed by SDS-PAGE and fluorography. (B and C) Phosphorimager analysis was performed to quantify the percentage of CA-SP1 relative to total CA-SP1 plus CA in pelleted virus samples. The data are plotted as the percentage of CA-SP1 in the presence of increasing concentrations of PF-46396 (B) or StA-MAT-23 (C). (D) The percentage of CA-SP1 in the presence of 1 μM PF-46396 was compared to the percentage of CA-SP1 in the presence of 1 μM PF-46396 plus increasing concentrations of StA-MAT-23. Statistical significance was assessed by using one-way analysis of variance with Dunnett's multiple-comparison posttest. *, *P* < 0.05. (E) The percentage of CA-SP1 in the presence of increasing concentrations of PF-46396 was compared with that in the presence of the equivalent concentration of PF-46396 plus 1 μM StA-MAT-23. Statistical significance was assessed by using one-way analysis of variance with Bonferroni's multiple-comparison posttest. *, *P* < 0.05; **, *P* < 0.01. Mean values from independent experiments are presented (*n* = 3), and error bars indicate standard deviations. Statistical analysis was preformed by using GraphPad Prism version 6 software.

We have clearly demonstrated that two inactive analogues (StA-MAT-23 and -30) are capable of interacting with WT Gag albeit with a reduced affinity compared to PF-46396. We next sought to determine if inactive analogue binding could interfere with PF-46396 binding and, hence, its activity. This is important because reduced levels of inactive analogue incorporation into WT particles may reflect binding to low-affinity sites on Gag rather than at the primary maturation inhibitor-binding site. To do this, we performed a competition assay with PF-46396 and StA-MAT-23 using various combinations of concentrations (Fig. 11). As expected, increasing concentrations of PF-46396 resulted in a dose-dependent increase in the amount of the uncleaved CA-SP1 intermediate (Fig. 11B). Increasing concentrations of StA-MAT-23 did not result in any significant accumulation of CA-SP1 (Fig. 11C). When the PF-46396 concentration remained constant at 1 μM, simultaneous treatment with increasing concentrations of StA-MAT-23 resulted in decreased CA-SP1 levels compared to treatment with 1 μM PF-46396 alone. This decrease in CA-SP1 levels was statistically significant when the highest concentration (5 μM) of StA-MAT-23 was tested (Fig. 11D). We next examined PF-46396 activity at various concentrations, either alone or in combination with 1 μM StA-MAT-23 (Fig. 11E). At all concentrations tested, the inhibitory activity of PF-46396 was significantly reduced when it was combined with 1 μM StA-MAT-23. Collectively, these data demonstrate that the inactive analogue StA-MAT-23 is able to interfere with the binding and, thus, the activity of PF-46396, suggesting that these two compounds compete for Gag binding, possibly at the same or an overlapping binding site(s).

## DISCUSSION

How chemically distinct pyridone-based (PF-46396) and betulinic acid-derived (BVM) HIV-1 maturation inhibitors apparently act by similar modes of action has yet to be determined. While structure-activity relationships of betulinic acid-derived compounds have been described previously (38, 46–48, 68, 69), the chemical determinants of PF-46396 antiviral activity have not been reported (35). In this study, we utilized a novel analogue series to examine PF-46396 structure-activity relationships with respect to compound antiviral activity and the ability to bind Gag.

We demonstrate that the *tert*-butyl group in the PF-46396 phenyl ring is essential for antiviral activity and cannot be replaced with a hydrogen, methyl, methoxy, trifluoromethyl, or chloro group. The presence of the *tert*-butyl group, however, is not an absolute requirement for Gag binding, because the inactive analogue StA-MAT-23, in which a methyl group replaces the *tert*-butyl group, is still incorporated relatively efficiently into WT immature particles. Indeed, StA-MAT-23 can antagonize PF-46396 activity, suggesting that these two compounds compete for Gag binding, possibly at the same or overlapping binding sites. Nonetheless, the contacts made by the *tert*-butyl group almost certainly improve the Gag binding efficiency (at least compared to the methyl group), because levels of incorporated StA-MAT-23 are ∼30% lower than those of PF-46396 in WT immature virus particles. Instead, our data suggest that the bulky *tert*-butyl group likely occupies space in the putative hydrophobic inhibitor-binding pocket such that its presence directly blocks PR access to the CA-SP1 cleavage site. Alternatively, the *tert*-butyl group may influence an allosteric site causing an alteration in the conformation, exposure, or flexibility of this region of Gag, such that PR cleavage of the CA-SP1 site is less efficient. Both of these possibilities were previously hypothesized to be modes of action utilized by maturation inhibitors (4, 27, 32, 58), and evidence that BVM binds Gag sequences directly overlapping the CA-SP1 cleavage site was generated by using photoaffinity analogues of BVM (58). Overall, StA-MAT-23 clearly demonstrates that a completely inactive analogue of PF-46363 is capable of some binding to WT immature virus particles, which is likely to be at or overlapping the primary maturation inhibitor-binding pocket in Gag. The implication of this observation is that at least some Gag binding may not be enough for inhibitor antiviral activity. Similarly, it has been proposed that binding of BVM to immature particles may not be sufficient for its antiviral activity, because particles assembled from Gag containing certain resistance mutations (CA-L231M or SP1-A1M) retain an intermediate capacity to incorporate and, hence, bind BVM (61).

The 2-aminoindan group of PF-46396 is important for antiviral activity, as the loss of this group (group 3 analogues) results in completely inactive compounds despite the presence of the *tert*-butyl group (StA-MAT-5). However, the presence of the 2-aminoindan group does not guarantee Gag binding because PF-4348182, which contains this group, was only very weakly incorporated into WT immature particles. PF-4348182 has both the *tert*-butyl and trifluoromethyl groups replaced, making assessments of the impact of the individual substitutions difficult. However, as discussed above, the *tert*-butyl group makes some contribution to WT Gag binding efficiency; therefore, it seems likely that replacement of the *tert*-butyl group by the 2-pyridyl group contributes to the loss of PF-4348182 binding. Despite the fact that the 2-aminoindan group makes a major contribution to the antiviral activity of PF-46396, its presence is not essential for Gag binding, as its replacement by an aminomethyl-cyclohexane (StA-MAT-30) results in an inactive analogue, which is incorporated into WT immature particles although less efficiently than PF-46396. Similarly, replacement by benzylamine (StA-MAT-18) leads to intermediate antiviral activity, and hence, a degree of Gag binding must occur for this analogue. However, the incorporation of a C_4_ substituent in the aryl ring of the benzylamine unit (StA-MAT-27, -28, and -29) abolishes any antiviral activity.

Assessment of the antiviral activities of analogues in which only the trifluoromethyl group has been changed (StA-MAT-17 and -21) showed that while this group appears optimal for antiviral activity, it is not essential because its replacement can be tolerated (by hydrogen or chlorine at least). The nonessential role of the trifluoromethyl group is related to antiviral activity in the context of WT Gag; however, this group appears to play a role in the compound-dependent rescue of the defective SP1-A3T HIV-1 mutant (see below). The optimal but nonessential role of the trifluoromethyl group in the context of WT Gag binding suggests that this site provides an opportunity to attach tags, such as an azido group, as utilized by Nguyen et al. (58), to facilitate detailed mapping of the PF-46396-binding site. This will allow further understanding of the relationship between antiviral activity and Gag binding and of how structurally distinct HIV-1 maturation inhibitors act by the same mode of action.

Overall, we have utilized this analogue series to dissect the contribution that different moieties of PF-46396 make toward antiviral activity and WT Gag binding and have started to determine the relationship between these two essential properties. Our analyses allow us to divide the analogues tested into three categories: (i) analogues that retained antiviral activity and, hence, WT Gag binding (StA-MAT-17 and -21); (ii) analogues that lost antiviral activity but retained some capacity to bind WT Gag (StA-MAT-23 and -30); and (iii) analogues that lost antiviral activity and the ability to bind WT Gag (PF-4348182 and StA-MAT-7). It seems likely that the inactive analogues that retain some capacity to bind WT Gag interact with the putative maturation inhibitor-binding site, as the analogue StA-MAT-23 interferes with PF-46396 activity, despite reduced levels of StA-MAT-23 incorporation into WT immature particles compared to PF-46396.

We also demonstrated that inactive analogues can be incorporated into mutant immature particles that are assembled from Gag molecules that contain the SP1-A3V mutation. When an analogue was incorporated into these mutant particles, it was independent of antiviral activity, as inactive compounds were incorporated as efficiently as PF-46396. Analogue incorporation into SP1-A3V immature particles correlated with their ability to rescue SP1-A3V replication in a compound-dependent manner. Compound dependency strongly suggests that rescue is facilitated by an analogue's capacity to interact with mutant Gag. Given that all analogues, with the exception of compounds in group 3, were capable of rescuing SP1-A3V, it is therefore reasonable to assume that these analogues are also capable of some interaction with SP1-A3V mutant Gag.

Interestingly, analogue incorporation into SP1-A3V immature particles was about half as efficient as PF-46396 incorporation into WT immature particles. At present, we cannot exclude the possibility that incorporation of compounds into SP1-A3V immature particles occurs at low-affinity sites in Gag that are unrelated to the maturation inhibitor-binding site. However, we think that this is unlikely, as the SP1-A3V mutation maps to the region of Gag that has been implicated in maturation inhibitor activity, and this mutation confers resistance to both PF-46396 and BVM (4, 39). Our data support the hypothesis that the mechanism of SP1-A3V resistance is not via the prevention of inhibitor binding, but instead, the mutation causes a conformational change in Gag that permits PR access to the CA-SP1 cleavage site even when the compound is bound (39). In addition, compound dependency reverses defective replication and particle production imposed on HIV-1 by the SP1-A3V mutation, implying that compound interaction directly influences the function of the CA-SP1 region of Gag to which the maturation inhibitor-binding pocket has been mapped. However, our data clearly suggest that the conformation of WT Gag is different from that of SP1-A3V Gag due to the differential incorporation of PF-46396, PF-4348182, StA-MAT-23, and StA-MAT-30 into immature particles assembled from each of these Gag molecules. While all of these compounds were incorporated as efficiently as each other into SP1-A3V particles (at least at 5 μM), variable incorporation into WT immature particles was observed, as analogues exhibited reduced (StA-MAT-23 and -30) or no (PF-4348182) incorporation compared to PF-46396.

Analogue incorporation into SP1-A3V particles appears to be highly flexible, as analogues with modifications of the 2-aminoindan, *tert*-butyl, and/or trifluoromethyl groups within PF-46396 were all tolerated. Indeed, as discussed above, it can be assumed that all analogues that rescue SP1-A3V in a compound-dependent manner are capable of some interaction with SP1-A3V Gag. The apparent plasticity of the compound-binding site created by the SP1-A3V mutation is not conferred by another maturation inhibitor resistance mutation, SP1-A3T, which maps to the same SP1 residue but imposes a more severe replication and particle production phenotype on HIV-1 (39). Rescue of SP1-A3T was confined to PF-46396 and just three analogues (StA-MAT-25, -18, and -30), and the presence of the trifluoromethyl group appeared to be necessary but not sufficient to rescue this mutant. Differential rescue of the CA MHR mutants was also observed in this study. However, the parental compound PF-46396 was capable of rescuing all the MHR and SP1 mutants studied. Overall, this suggests that an analogue's capacity to rescue defective HIV-1 can be influenced by the effect of the mutation in Gag and/or the chemical structure of the analogue. Unfortunately, the lack of atomic-resolution structural information for the CA-SP1 region of Gag (17, 27) makes it challenging to interpret why a particular combination of a mutation and an analogue facilitates rescue.

In conclusion, we have utilized a series of analogues to determine PF-46396 structure-activity relationships with respect to antiviral activity and binding to both WT and mutant Gag proteins. The lack of a correlation between an analogue's ability to bind Gag and its antiviral activity enabled us to start to dissect the contribution that different components of the PF-46396 structure make to each of these essential roles and/or the relationship between them.

## Supplementary Material

Supplemental material
