# Supplementary material for: Structure-Activity Relationships of the Human Immunodeficiency Virus Type 1 Maturation Inhibitor PF-46396
Source: J Virol. 2016 Aug 26;90(18):8181–97. doi: 10.1128/JVI.01075-16 (PMC5008107; doi:10.1128/JVI.01075-16)
Supplement: Supplemental material [file supp_90_18_8181__index.html]

Structure-Activity Relationships of the Human Immunodeficiency Virus Type 1 Maturation Inhibitor PF-46396 — Supplemental material 

# Structure-Activity Relationships of the Human Immunodeficiency Virus Type 1 Maturation Inhibitor PF-46396

## Supplemental material

- Supplemental file 1 -

  Fig. S1 (PF-46396 analogue synthesis and analysis.)

  PDF, 253K
