# Supplementary material for: Structure-Activity Relationships of the Human Immunodeficiency Virus Type 1 Maturation Inhibitor PF-46396
Source: J Virol. 2016 Aug 26;90(18):8181–97. doi: 10.1128/JVI.01075-16 (PMC5008107; doi:10.1128/JVI.01075-16)
Supplement: Supplemental material [file JVI.01075-16_zjv999181918so1.pdf]

Supplementary Fig. 1. PF-46396 analogue synthesis and analysis

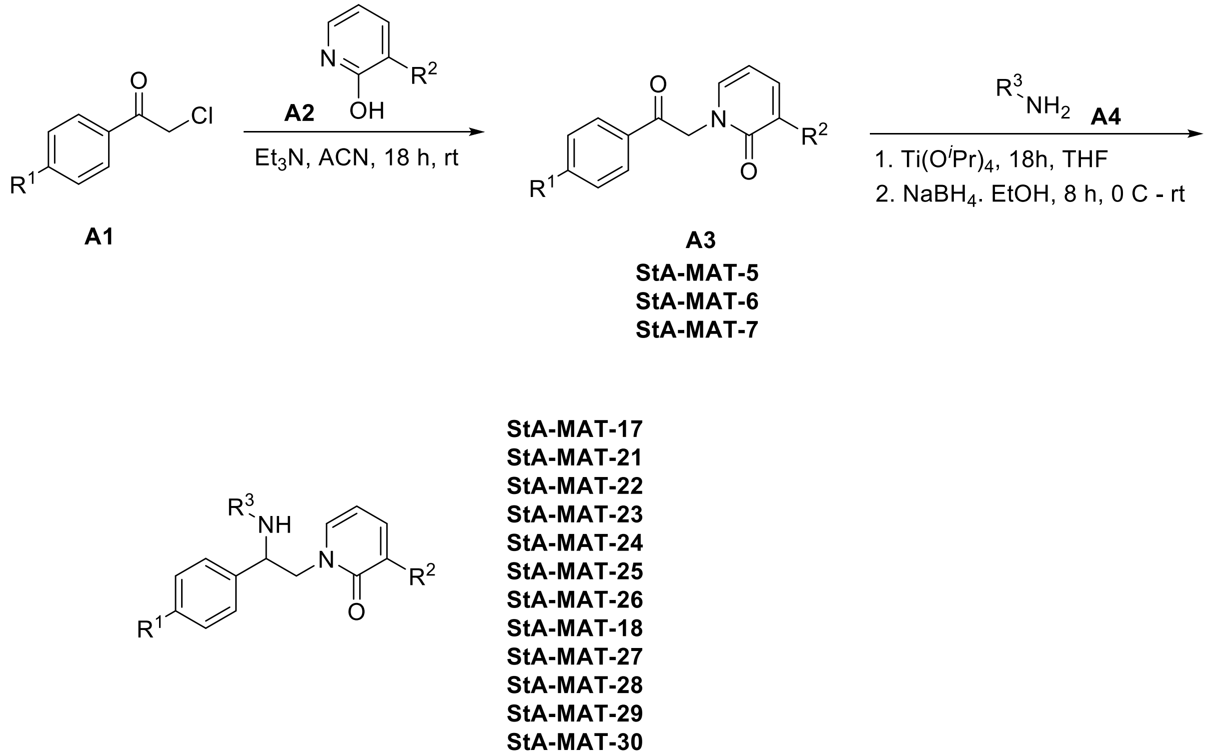

Scheme A1: Synthetic route to **StA-MAT** analogues

General Procedure 1

To a solution of the pyridine **A2** (1 g) in anhydrous acetonitrile (120 mL) was added the acetophenone **A1** (4 eq.) followed by triethylamine (5 eq.). The resulting mixture was then stirred at room temperature for 18 h and the solvent removed *in vacuo*. The residue obtained was then purified by column chromatography (DCM) to yield the desired product **A3** or **StA-MAT-5** or **StA-MAT-6** or **StA-MAT-7**.

General Procedure 2

To a solution of **A3** or **StA-MAT-5 StA-MAT-6** or **StA-MAT-7** (1.2 eq.) in anhydrous THF (70 mL) was added titanium *iso*propoxide (2 eq.) followed by amine **A4** (500 mg). The resulting brown solution was then stirred at room temperature for 18 hours. A solution of $NaBH_4$ (3 eq.) in ethanol (15 mL) was added at 0 °C and the resulting mixture allowed to warm up to room temperature. It was then stirred at room temperature for an additional 8h before the solvent was removed *in vacuo*. The crude product obtained was purified by column chromatography (0 % to 1 % MeOH in DCM) to yield the desired product.

These general procedures have been used to synthesise all compounds described and tested in this work. As an example, details are provided for the synthesis of **A3** (with R^1 = ^{t}Bu and R^2 = H) and **StA-MAT-21**.

1-(2-(4-(*tert*-Butyl)phenyl)-2-oxoethyl)pyridin-2(1*H*)-one

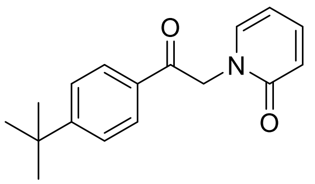

A3 with R^1 = ^{t}Bu and R^2 = H

To a solution of the 2-hydroxypyridine **A2** (with R^2 = H) (1 g) in anhydrous acetonitrile (120 mL) was added the 4'-*tert*butyl-2-chloroacetophenone **A3** (with R^1 = ^{t}Bu) (4 eq.) followed by triethylamine (5 eq.). The resulting mixture was then stirred at room temperature for 18 h and the solvent removed *in vacuo*. The residue obtained was then purified by column chromatography (DCM) to yield the desired product **A3** (with R^1 = ^{t}Bu and R^2 = H) (115 mg, 39%). ^{1}H NMR (400 MHz, MeOD) δ 8.07 – 7.99 (m, 2H), 7.66 – 7.56 (m, 4H), 6.64 – 6.56 (m, 1H), 6.49 – 6.42 (m, 1H), 5.54 (s, 2H), 1.39 (s, 9H); ^{13}C NMR (126 MHz, MeOD) δ 192.0, 163.4, 157.7, 141.3, 139.5, 132.2, 127.8, 125.6, 125.5, 119.1, 107.0, 55.1, 30.0; HR-MS (ESI) m/z calculated for $C_{17}H_{19}NO_2Na$ [M+Na]$^+$, expected 292.1308; found 292.1304.

1-(2-(4-(*tert*-Butyl)phenyl)-2-((2,3-dihydro-1*H*-inden-2-yl)amino)ethyl)pyridin-2(1*H*)-one hydrochloride

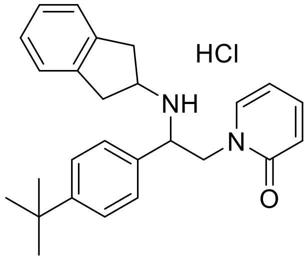

StA-MAT-21

To a solution of **A3** (with R^1 = ^{t}Bu and R^2 = H) (1.2 eq.) in anhydrous THF (70 mL) was added titanium *iso*propoxide (2 eq.) followed by 2-aminoindane **A4** (R^3 = indane) (500 mg). The resulting brown solution was then stirred at room temperature for 18 hours. A solution of $NaBH_4$ (3 eq.) in ethanol (15 mL) was added at 0 °C and the resulting mixture allowed to warm up to room temperature. It was then stirred at room temperature for an additional 8h before the solvent was removed *in vacuo*. The crude product obtained was purified by column chromatography (0 % to 1 % MeOH in DCM) to yield the desired product **StA-MAT-21** (43 mg, 46%). ^{1}H NMR (500 MHz, MeOD) δ 7.57 – 7.44 (m, 5H), 7.29 – 7.14 (m, 5H), 6.58 (d, *J* = 8.9 Hz, 1H), 6.21 – 6.15 (m, 1H), 5.05 – 4.76 (m, 2H), 4.46 – 4.38 (m, 1H), 4.06 – 3.99 (m, 1H), 3.44 – 3.34 (m, 2H), 3.27 – 3.19 (m, 1H), 3.14 (dd, *J* = 16.1, 6.5 Hz, 1H), 1.33 (s, 9H); ^{13}C NMR (126 MHz, MeOD) δ 163.9, 153.6, 141.6, 139.0, 138.9, 138.5, 138.3, 128.0, 127.2, 126.4, 124.3, 124.2, 119.4, 107.3, 59.1, 56.7, 52.3, 36.1, 35.4, 30.2; HR-MS (ESI) m/z calculated for $C_{27}H_{31}N_2O$ [M+H]$^+$, expected 387.2431; found 387.2429.

Experimental Procedures: Group 1 Analogues

6-(2-(4-(*tert*-Butyl)phenyl)-2-((2,3-dihydro-1*H*-inden-2-yl)amino)ethyl)-2-(trifluoromethyl) cyclohexa-2,4-dien-1-one hydrochloride

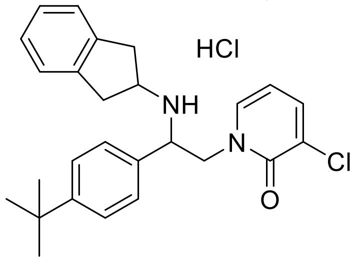

StA-MAT-17

StA-MAT-17 (62 mg, 38%) was obtained following general procedure 2 using **A3** (with R^1 = ^{t}Bu and R^2 = Cl) and 2-aminoindane. ^{1}H NMR (500 MHz, DMSO-*d6*) δ 10.21 (s, 1H), 10.05 (s, 1H), 7.82 (t, *J* = 7.9 Hz, 1H), 7.69 (d, *J* = 8.1 Hz, 2H), 7.52 (d, *J* = 8.0 Hz, 2H), 7.27 – 7.14 (m, 5H), 6.93 (d, *J* = 8.2 Hz, 1H), 4.88 – 4.77 (m, 2H), 4.69 (dd, *J* = 10.9, 4.6 Hz, 1H), 3.90 – 3.80 (m, 1H), 3.31 – 3.17 (m, 4H), 1.29 (s, 9H); ^{13}C NMR (126 MHz, DMSO-*d6*) δ 162.4, 152.5, 147.5, 143.0, 139.9, 139.6, 130.5, 128.9, 127.5, 126.4, 124.9, 117.9, 110.2, 66.7, 59.0, 56.2, 36.3, 35.7, 34.9, 31.5; HR-MS (ESI) m/z calculated for $C_{26}H_{30}{}^{35}ClN_2O$ [M+H]$^+$, expected 421.2041; found 421.2038.

1-(2-((2,3-Dihydro-1*H*-inden-2-yl)amino)-2-(*p*-tolyl)ethyl)-3-(trifluoromethyl)pyridin-2(1*H*)-one hydrochloride

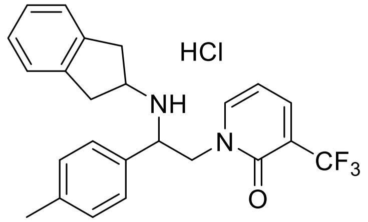

StA-MAT-23

StA-MAT-23 (45 mg, 64%) was obtained following general procedure 2 using **A3** (with R^1 = CH_3 and R^2 = CF_3) and 2-aminoindane. ^{1}H NMR (500 MHz, MeOD) δ 7.90 (dd, *J* = 7.1, 1.6 Hz, 1H), 7.44 – 7.37 (m, 3H), 7.36 – 7.18 (m, 6H), 6.24 (t, *J* = 7.0 Hz, 1H), 4.97 – 4.91 (m, 1H), 4.86 (dd, *J* = 8.5, 5.1 Hz, 1H), 4.37 (dd, *J* = 13.1, 8.6 Hz, 1H), 4.04 (p, *J* = 7.4 Hz, 1H), 3.43 - 3.34 (m, 2H), 3.22 (dd, *J* = 16.3, 7.1 Hz, 1H), 3.10 (dd, *J* = 16.1, 7.0 Hz, 1H), 2.38 (s, 3H); ^{13}C NMR (126 MHz, MeOD) δ 159.1, 143.2, 140.7, 140.4 (q, *J* = 4.8 Hz), 138.6, 138.4, 130.1, 128.0, 127.2, 124.3, 124.3, 121.6, 119.4 (assigned by HMBC), 104.9, 58.5, 56.8, 52.0, 36.2, 35.6, 19.8; HR-MS (ESI) m/z calculated for $C_{24}H_{24}F_3N_2O$ [M+H]$^+$, expected 413.1835; found 413.111832.

1-(2-((2,3-Dihydro-1*H*-inden-2-yl)amino)-2-phenylethyl)-3-(trifluoromethyl)pyridin-2(1*H*)-one hydrochloride

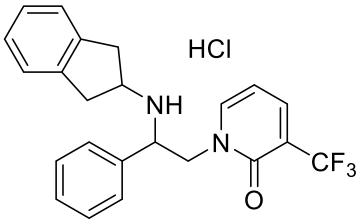

StA-MAT-22

StA-MAT-22 (31 mg, 23%) was obtained following general procedure 2 using **A3** (with R^1 = H and R^2 = CF_3) and 2-aminoindane. ^{1}H NMR (400 MHz, MeOD) δ 7.88 (d, *J* = 7.2 Hz, 1H), 7.52 – 7.46 (m, 5H), 7.40 (d, *J* = 6.9 Hz, 1H), 7.27 – 7.16 (m, 4H), 6.21 (t, *J* = 7.0 Hz, 1H), 4.36 (dd, *J* = 12.5, 7.7 Hz, 1H), 4.02 – 3.97 (m, 1H), 3.41 – 3.29 (m, 4H), 3.22 – 3.12 (m, 1H), 3.06 (dd, *J* = 16.1, 7.1 Hz, 1H); HR-MS (ESI) m/z calculated for $C_{23}H_{22}F_3N_2O$ [M+H]$^+$, expected 399.1679; found 399.1672.

1-(2-((2,3-Dihydro-1*H*-inden-2-yl)amino)-2-(4-methoxyphenyl)ethyl)-3-(trifluoromethyl)pyridin-2(1*H*)-one hydrochloride

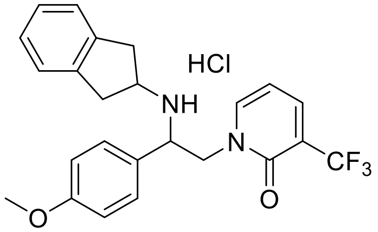

StA-MAT-24

StA-MAT-24 (39 mg, 52%) was obtained following general procedure 2 using **StA-MAT-6** and 2-aminoindane. ^{1}H NMR (400 MHz, DMSO-*d6*) δ 10.22 (s, 1H), 9.97 (s, 1H), 7.88 (d, *J* = 7.1 Hz, 1H), 7.68 (d, *J* = 6.1 Hz, 1H), 7.57 (d, *J* = 8.4 Hz, 2H), 7.25 – 7.13 (m, 4H), 7.06 – 6.95 (m, 2H), 6.22 (t, *J* = 7.0 Hz, 1H), 4.84 (dd, *J* = 12.9, 5.7 Hz, 1H), 4.77 – 4.72 (m, 1H), 4.36 (dd, *J* = 12.9, 8.6 Hz, 1H), 3.82 – 3.72 (m, 4H), 3.25 – 3.05 (m, 4H); HR-MS (ESI) m/z calculated for $C_{24}H_{24}F_3N_2O_2$ [M+H]$^+$, expected 429.1784; found 429.1781.

1-(2-((2,3-Dihydro-1*H*-inden-2-yl)amino)-2-(4-(trifluoromethyl)phenyl)ethyl)-3-(trifluoromethyl) pyridine-2(1*H*)-one hydrochloride

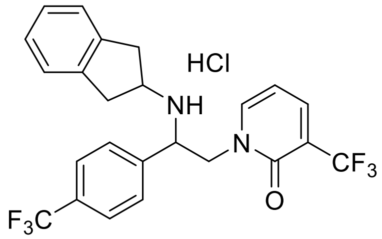

StA-MAT-25

StA-MAT-25 (14 mg, 57%) was obtained following general procedure 2 using **A3** (with $R^1 = CF_3$ and $R^2 = CF_3$) and 2-aminoindane. ^1H NMR (400 MHz, MeOD) δ 7.92 (d, *J* = 7.4 Hz, 1H), 7.83 (d, *J* = 8.1 Hz, 2H), 7.72 (d, *J* = 8.1 Hz, 2H), 7.47 (d, *J* = 7.0 Hz, 1H), 7.29 – 7.18 (m, 4H), 6.28 (t, *J* = 7.0 Hz, 1H), 5.02 – 4.97 (m, 1H), 4.46 (dd, *J* = 13.4, 8.1 Hz, 1H), 4.21 – 3.96 (m, 1H), 3.59 – 2.90 (m, 5H); HR-MS (ESI) m/z calculated for $C_{24}H_{21}F_6N_2O$ $[M+H]^+$, expected 467.1553; found 467.1547.

1-(2-(4-Chlorophenyl)-2-((2,3-dihydro-1*H*-inden-2-yl)amino)ethyl)-3-(trifluoromethyl)pyridin-2(1*H*)-one hydrochloride

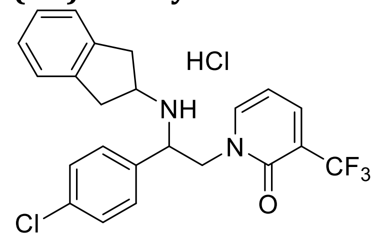

StA-MAT-26

StA-MAT-26 (34 mg, 46%) was obtained following general procedure 2 using **StA-MAT-7** and 2-aminoindane. ^1H NMR (700 MHz, MeOD) δ 7.91 (d, *J* = 6.9 Hz, 1H), 7.53 (s, 4H), 7.46 (d, *J* = 6.4 Hz, 1H), 7.27 (dt, *J* = 13.4, 3.6 Hz, 2H), 7.24 – 7.20 (m, 2H), 6.28 (t, *J* = 6.8 Hz, 1H), 4.98 – 4.92 (m, 2H), 4.45 – 4.40 (m, 1H), 4.13 – 4.08 (m, 1H), 3.45 – 3.36 (m, 2H), 3.24 (dd, *J* = 16.1, 6.8 Hz, 1H), 3.13 (dd, *J* = 16.1, 6.7 Hz, 1H); ^{13}C NMR (126 MHz, MeOD) δ 159.1, 143.2, 140.4 (q, *J* = 4.7 Hz), 138.7, 135.8, 129.8, 129.5, 127.0, 124.2, 123.8, 121.6, 119.6 (assigned by HMBC), 105.0, 58.1, 56.9, 52.2, 36.7, 36.0; HR-MS (ESI) m/z calculated for $C_{23}H_{21}{}^{35}ClF_3N_2O$ $[M+H]^+$, expected 433.1289; found 433.1286.

<u>Experimental Procedures: Group 2 Analogues</u>

1-(2-(Benzylamino)-2-(4-(*tert*-butyl)phenyl)ethyl)-3-(trifluoromethyl)pyridin-2(1*H*)-one hydrochloride

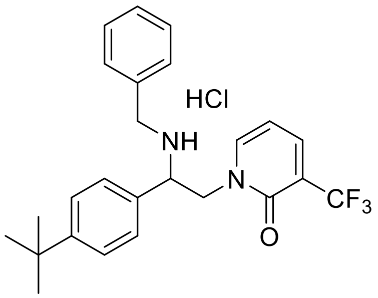

StA-MAT-18

StA-MAT-18 (81 mg, 17%) was obtained following general procedure 2 using **StA-MAT-5** and benzylamine. ^{1}H NMR (400 MHz, MeOD) δ 7.89 (ddd, *J* = 7.2, 2.0, 0.9 Hz, 1H), 7.59 – 7.51 (m, 2H), 7.49 – 7.35 (m, 8H), 6.29 – 6.20 (m, 1H), 4.94 – 4.90 (m, 1H), 4.81 (dd, *J* = 7.4, 5.9 Hz, 1H), 4.41 (dd, *J* = 13.3, 7.4 Hz, 1H), 4.22 (d, *J* = 13.1 Hz, 1H), 4.08 (d, *J* = 13.1 Hz, 1H), 1.33 (s, 9H); ^{13}C NMR (126 MHz, MeOD) δ 159.3, 153.7, 143.1, 140.4 (q, *J* = 5.2 Hz), 130.8, 129.7, 129.4, 128.9, 127.9, 126.5, 123.8, 121.6, 119.4 (q, *J* = 31.1 Hz), 105.0, 59.9, 52.1, 49.9, 34.3, 30.1; HR-MS (ESI) m/z calculated for $C_{25}H_{28}F_3N_2O$ $[M+H]^+$, expected 429.2148; found 429.2146.

1-(2-(4-(*tert*-Butyl)phenyl)-2-((cyclohexylmethyl)amino)ethyl)-3-(trifluoromethyl)pyridin-2(1*H*)-one hydrochloride

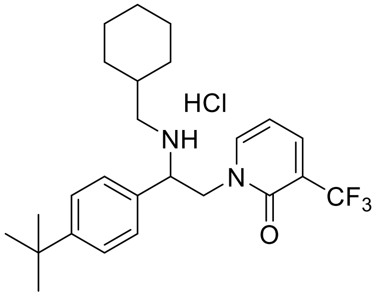

StA-MAT-30

StA-MAT-30 (42 mg, 60%) was obtained following general procedure 2 using **StA-MAT-5** and cyclohexylbenzylamine. ^{1}H NMR (500 MHz, MeOD) δ 7.95 – 7.89 (m, 1H), 7.57 – 7.51 (m, 2H), 7.47 (dd, *J* = 6.8, 2.0 Hz, 1H), 7.41 – 7.34 (m, 2H), 6.28 (t, *J* = 7.0 Hz, 1H), 4.72 (dd, *J* = 7.4, 5.7 Hz, 1H), 4.40 (dd, *J* = 13.6, 7.4 Hz, 1H), 2.93 (dd, *J* = 12.5, 7.0 Hz, 1H), 2.73 (dd, *J* = 12.5, 7.0 Hz, 1H), 1.85 – 1.67 (m, 6H), 1.39 – 1.19 (m, 12H), 1.09 – 0.95 (m, 2H); ^{13}C NMR (126 MHz, MeOD) δ 159.4, 153.6, 143.2, 140.4 (q, *J* = 4.8 Hz), 127.7, 126.4, 123.8, 121.6, 105.1, 60.7, 52.5, 52.2, 48.1, 35.1, 34.3, 30.1, 25.5, 25.1; HR-MS (ESI) m/z calculated for $C_{25}H_{34}F_3N_2O$ $[M+H]^+$, expected 435.2618; found 435.2616.

1-(2-(4-(*tert*-Butyl)phenyl)-2-((4-methoxybenzyl)amino)ethyl)-3-(trifluoromethyl)pyridin-2(1*H*)-one hydrochloride

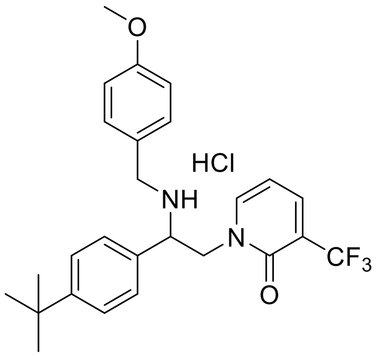

StA-MAT-27

StA-MAT-27 (35 mg, 48%) was obtained following general procedure 2 using **StA-MAT-5** and 4-methoxybenzylamine. ^{1}H NMR (400 MHz, DMSO-*d6*) δ 10.10 (s, 1H), 9.80 (s, 1H), 7.88 (d, *J* = 7.2 Hz, 1H), 7.71 (s, 1H), 7.53 – 7.41 (m, 4H), 7.37 (d, *J* = 8.3 Hz, 2H), 6.94 (d, *J* = 8.2 Hz, 2H), 6.22 (t, *J* = 7.0 Hz, 1H), 4.88 – 4.83 (m, 1H), 4.76 – 4.71 (m, 1H), 4.32 – 4.27 (m, 2H), 4.03 – 3.98 (m, 1H), 3.75 (s, 3H), 1.26 (s, 9H); HR-MS (ESI) m/z calculated for $C_{26}H_{30}F_3N_2O_2$ $[M+H]^+$, expected 459.2254; found 459.2248.

1-(2-(4-(*tert*-Butyl)phenyl)-2-((4-(trifluoromethyl)benzyl)amino)ethyl)-3-(trifluoromethyl)pyridin-2(1*H*)-one hydrochloride

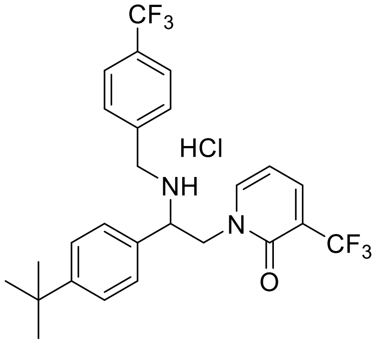

StA-MAT-28

StA-MAT-28 (41 mg, 52%) was obtained following general procedure 2 using **StA-MAT-5** and 4-(trifluoromethyl) benzylamine. ^{1}H NMR (400 MHz, MeOD) δ 7.90 (ddd, *J* = 7.3, 2.0, 0.9 Hz, 1H), 7.78 – 7.74 (m, 2H), 7.66 (d, *J* = 8.1 Hz, 2H), 7.59 – 7.50 (m, 2H), 7.50 – 7.36 (m, 3H), 6.30 – 6.22 (m, 1H), 4.98 – 4.81 (m, 2H), 4.50 – 4.32 (m, 2H), 4.20 (d, *J* = 13.3 Hz, 1H), 1.33 (s, 9H); HR-MS (ESI) m/z calculated for $C_{26}H_{27}F_6N_2O$ $[M+H]^+$, expected 497.2022; found 497.2019.

1-(2-((4-Bromobenzyl)amino)-2-(4-(*tert*-butyl)phenyl)ethyl)-3-(trifluoromethyl)pyridin-2(1*H*)-one hydrochloride

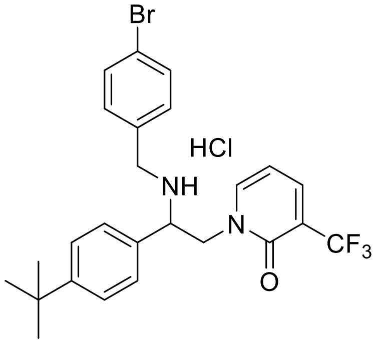

StA-MAT-29

StA-MAT-29 (21 mg, 26%) was obtained following general procedure 2 using **StA-MAT-5** and 4-bromoaniline. ^{1}H NMR (400 MHz, MeOD) δ 7.90 (ddd, *J* = 7.1, 2.0, 0.9 Hz, 1H), 7.64 – 7.59 (m, 2H), 7.59 – 7.51 (m, 2H), 7.49 – 7.34 (m, 5H), 6.30 – 6.22 (m, 1H), 4.97 – 4.90 (m, 1H), 4.86 – 4.80 (m, 1H), 4.43 (d, *J* = 7.4 Hz, 1H), 4.29 – 4.20 (m, 1H), 4.10 (d, *J* = 13.2 Hz, 1H), 1.34 (s, 9H); HR-MS (ESI) m/z calculated for $C_{25}H_{27}{}^{79}BrF_3N_2O$ $[M+H]^+$, expected 507.1253; found 507.1246.

Experimental Procedures: Group 3 Analogues

1-(2-(4-Chlorophenyl)-2-oxoethyl)-3-(trifluoromethyl)pyridin-2(1*H*)-one

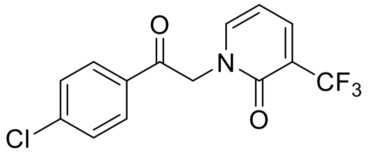

StA-MAT-7

StA-MAT-7 (201 mg, 41%) was obtained following general procedure 1 using 2,4'-dichloroacetophenone and 2-hydroxy-3-(trifluoromethyl) pyridine. ^{1}H NMR (500 MHz, MeOD) δ 8.13 – 8.07 (m, 2H), 8.07 – 8.00 (m, 1H), 7.88 (dd, *J* = 6.8, 1.9 Hz, 1H), 7.65 – 7.57 (m, 2H), 6.53 (t, *J* = 7.0 Hz, 1H), 5.59 (s, 2H); ^{13}C NMR (126 MHz, MeOD) δ 158.8, 143.8, 140.2 (q, *J* = 4.9 Hz)), 140.1, 133.2, 129.5, 128.9, 124.0, 121.8, 119.4 (q, *J* = 31.2 Hz), 104.8, 55.1; HR-MS (ESI) m/z calculated for $C_{14}H_{10}{}^{35}ClF_3NO_2$ $[M+H]^+$, expected 316.0347; found 316.0344.

1-(2-(4-(*tert*-Butyl)phenyl)-2-oxoethyl)-3-(trifluoromethyl)pyridin-2(1*H*)-one

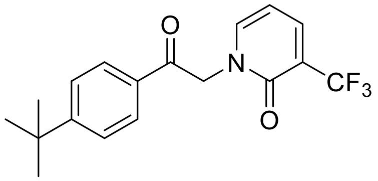

StA-MAT-5

StA-MAT-5 (950 mg, 46%) was obtained following general procedure 1 using 4'-*tert*butyl-2-chloroacetophenone and 2-hydroxy-3-(trifluoromethyl) pyridine. ^{1}H NMR (400 MHz, $CDCl_3$) δ 8.03 – 7.95 (m, 2H), 7.84 (ddt, *J* = 7.2, 2.1, 0.9 Hz, 1H), 7.60 – 7.51 (m, 2H), 7.48 (ddt, *J* = 6.8, 2.1, 0.6 Hz, 1H), 6.35 (ddd, *J* = 7.1, 6.7, 0.7 Hz, 1H), 5.43 (s, 2H), 1.38 (s, 9H); ^{13}C NMR (126 MHz, $CDCl_3$) δ 191.3, 158.3, 142.7, 139.4 (q, *J* = 5.0 Hz), 131.8, 128.2, 126.0, 123.7, 121.6, 120.5, 119.4 (q, *J* = 31.0 Hz), 104.1, 54.0, 31.0; HR-MS (ESI) m/z calculated for $C_{18}H_{18}NO_2Na$ $[M+Na]^+$, expected 360.1182; found 360.1176.

1-(2-(4-Methoxyphenyl)-2-oxoethyl)-3-(trifluoromethyl)pyridin-2(1*H*)-one

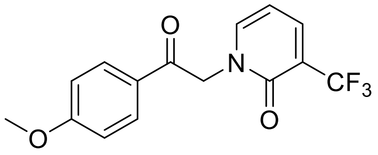

StA-MAT-6

StA-MAT-6 (215 mg, 45%) was obtained following general procedure 1 using 4'-methoxy-2-chloroacetophenone and 2-hydroxy-3-(trifluoromethyl) pyridine. ^{1}H NMR (400 MHz, $CDCl_3$) δ 8.07 – 7.98 (m, 2H), 7.82 (ddt, *J* = 7.2, 2.0, 0.9 Hz, 1H), 7.49 (ddt, *J* = 6.8, 2.1, 0.7 Hz, 1H), 7.04 – 6.95 (m, 2H), 6.33 (ddt, *J* = 7.2, 6.6, 0.7 Hz, 1H), 5.39 (s, 2H), 3.91 (s, 3H); HR-MS (ESI) m/z calculated for $C_{15}H_{12}NO_3Na$ $[M+Na]^+$, expected 334.0661; found 334.0657.
